# Analysis of Dual Combination Therapies Used in Treatment of Hypertension in a Multinational Cohort

**DOI:** 10.1001/jamanetworkopen.2022.3877

**Published:** 2022-03-24

**Authors:** Yuan Lu, Mui Van Zandt, Yun Liu, Jing Li, Xialin Wang, Yong Chen, Zhengfeng Chen, Jaehyeong Cho, Sreemanee Raaj Dorajoo, Mengling Feng, Min-Huei Hsu, Jason C. Hsu, Usman Iqbal, Jitendra Jonnagaddala, Yu-Chuan Li, Siaw-Teng Liaw, Hong-Seok Lim, Kee Yuan Ngiam, Phung-Anh Nguyen, Rae Woong Park, Nicole Pratt, Christian Reich, Sang Youl Rhee, Selva Muthu Kumaran Sathappan, Seo Jeong Shin, Hui Xing Tan, Seng Chan You, Xin Zhang, Harlan M. Krumholz, Marc A. Suchard, Hua Xu

**Affiliations:** 1 Center for Outcomes Research and Evaluation, Yale New Haven Hospital, New Haven, Connecticut; 2 Section of Cardiovascular Medicine, Department of Internal Medicine, Yale School of Medicine, New Haven, Connecticut; 3 Real World Solutions, Iqvia, Durham, North Carolina; 4 School of Biomedical Engineering and Informatics, Department of Medical Informatics, Nanjing Medical University, Jiangsu, China; 5 Perelman School of Medicine, Department of Biostatistics, Epidemiology and Informatics, University of Pennsylvania; 6 National University Heart Center, Yong Loo Lin School of Medicine, National University of Singapore, Singapore; 7 Department of Biomedical Sciences, Ajou University Graduate School of Medicine, Suwon, Republic of Korea; 8 Health Sciences Authority, Singapore and Khoo Teck Puat Hospital, Singapore; 9 Saw Swee Hock School of Public Health, National University of Singapore, Singapore; 10 Institute of Data Science, National University of Singapore, Singapore; 11 Taipei Medical University, Taipei, Taiwan; 12 International PhD Program in Biotech and Healthcare Management, College of Management, Taipei Medical University, Taipei, Taiwan; 13 International Center for Health Information Technology, Taipei Medical University, Taipei City, Taiwan; 14 World Health Organization Collaborating Center on eHealth, School of Population Health, University of New South Wales Sydney, Australia; 15 Graduate Institute of Biomedical Informatics, College of Medical Science and Technology, Taipei Medical University, Taipei City, Taiwan; 16 Department of Cardiology, Ajou University School of Medicine, Suwon, Republic of Korea; 17 Group Chief Technology Office, National University Health System, Singapore; 18 International Center for Health Information Technology, Taipei Medical University, Taipei, Taiwan; 19 School of Health Technology, Taiwan Department of Healthcare Information and Management, Ming Chuan University, Taipei, Taiwan; 20 Department of Biomedical Informatics, Ajou University School of Medicine, Suwon, Republic of Korea; 21 Department of Biomedical Sciences, Ajou University Graduate School of Medicine, Suwon, Republic of Korea; 22 Quality Use of Medicines and Pharmacy Research Center, Clinical and Health Sciences, University of South Australia, Adelaide, Australia; 23 Real World Solutions, Iqvia, Cambridge, Massachusetts; 24 Kyung Hee University Medical Center, Seoul, Republic of Korea; 25 National University of Singapore, Singapore; 26 Health Sciences Authority, Singapore; 27 Department of Preventive Medicine, Yonsei University College of Medicine, Seoul, Republic of Korea; 28 Department of Health Policy and Management, Yale School of Public Health, New Haven, Connecticut; 29 Department of Biostatistics, Fielding School of Public Health, University of California, Los Angeles; 30 University of Texas Health Science Center at Houston, Houston

## Abstract

**Importance:**

More than 1 billion adults have hypertension globally, of whom 70% cannot achieve their hypertension control goal with monotherapy alone. Data are lacking on clinical use patterns of dual combination therapies prescribed to patients who escalate from monotherapy.

**Objective:**

To investigate the most common dual combinations prescribed for treatment escalation in different countries and how treatment use varies by age, sex, and history of cardiovascular disease.

**Design, Setting, and Participants:**

This cohort study used data from 11 electronic health record databases that cover 118 million patients across 8 countries and regions between January 2000 and December 2019. Included participants were adult patients (ages ≥18 years) who newly initiated antihypertensive dual combination therapy after escalating from monotherapy. There were 2 databases included for 3 countries: the Iqvia Longitudinal Patient Database (LPD) Australia and Electronic Practice-based Research Network 2019 linked data set from South Western Sydney Local Health District (ePBRN SWSLHD) from Australia, Ajou University School of Medicine (AUSOM) and Kyung Hee University Hospital (KHMC) databases from South Korea, and Khoo Teck Puat Hospital (KTPH) and National University Hospital (NUH) databases from Singapore. Data were analyzed from June 2020 through August 2021.

**Exposures:**

Treatment with dual combinations of the 4 most commonly used antihypertensive drug classes (angiotensin-converting enzyme inhibitor [ACEI] or angiotensin receptor blocker [ARB]; calcium channel blocker [CCB]; β-blocker; and thiazide or thiazide-like diuretic).

**Main Outcomes and Measures:**

The proportion of patients receiving each dual combination regimen, overall and by country and demographic subgroup.

**Results:**

Among 970 335 patients with hypertension who newly initiated dual combination therapy included in the final analysis, there were 11 494 patients from Australia (including 9291 patients in Australia LPD and 2203 patients in ePBRN SWSLHD), 6980 patients from South Korea (including 6029 patients in Ajou University and 951 patients in KHMC), 2096 patients from Singapore (including 842 patients in KTPH and 1254 patients in NUH), 7008 patients from China, 8544 patients from Taiwan, 103 994 patients from France, 76 082 patients from Italy, and 754 137 patients from the US. The mean (SD) age ranged from 57.6 (14.8) years in China to 67.7 (15.9) years in the Singapore KTPH database, and the proportion of patients by sex ranged from 24 358 (36.9%) women in Italy to 408 964 (54.3%) women in the US. Among 12 dual combinations of antihypertensive drug classes commonly used, there were significant variations in use across country and patient subgroup. For example starting an ACEI or ARB monotherapy followed by a CCB (ie, ACEI or ARB + CCB) was the most commonly prescribed combination in Australia (698 patients in ePBRN SWSLHD [31.7%] and 3842 patients in Australia LPD [41.4%]) and Singapore (216 patients in KTPH [25.7%] and 439 patients in NUH [35.0%]), while in South Korea, CCB + ACEI or ARB (191 patients in KHMC [20.1%] and 1487 patients in Ajou University [24.7%]), CCB + β-blocker (814 patients in Ajou University [13.5%] and 217 patients in KHMC [22.8%]), and ACEI or ARB + CCB (147 patients in KHMC [15.5%] and 1216 patients in Ajou University [20.2%]) were the 3 most commonly prescribed combinations. The distribution of 12 dual combination therapies were significantly different by age and sex in almost all databases. For example, use of ACEI or ARB + CCB varied from 873 of 3737 patients ages 18 to 64 years (23.4%) to 343 of 2292 patients ages 65 years or older (15.0%) in South Korea’s Ajou University database (*P* for database distribution by age < .001), while use of ACEI or ARB + CCB varied from 2121 of 4718 (44.8%) men to 1721 of 4549 (37.7%) women in Australian LPD (*P* for drug combination distributions by sex < .001).

**Conclusions and Relevance:**

In this study, large variation in the transition between monotherapy and dual combination therapy for hypertension was observed across countries and by demographic group. These findings suggest that future research may be needed to investigate what dual combinations are associated with best outcomes for which patients.

## Key Points

**Question:**

What are the most common antihypertensive dual combinations prescribed to patients who escalate from monotherapy in clinical practice, and how do the combinations differ by country and patient demographic subgroup?

**Findings:**

In this cohort study of 970 335 individuals from 11 large databases, 12 dual combinations of antihypertensive drug classes were commonly used, with large variation across countries and demographic groups.

**Meaning:**

These findings on the diversity of approaches used in practice suggest that future research is needed to investigate what medication combinations are associated with best outcomes for which patients.

## Teaser

This cohort study investigates differences in dual combination therapy use for hypertension in 8 countries and territories by country and patient demographic group.

## Introduction

Hypertension is the leading global risk factor associated with cardiovascular disease (CVD) and chronic kidney disease, contributing worldwide to more than 7 million deaths and 57 million disability-adjusted life-years annually.^1^ In 2015, approximately 1.13 billion adults had hypertension, yet fewer than 30% had achieved their blood pressure control goal.^2^ Notably, the burden of hypertension is particularly salient in the Asia Pacific region, given that this region has 60% of the world’s population and has experienced a rapid increase in prevalence of hypertension since 1980.^2,3^

Approximately 70% of patients with hypertension cannot achieve their blood pressure control goal with monotherapy.^4^ Despite the wide availability of antihypertensive agents, considerable uncertainty remains regarding the optimal choice for a second agent for use in escalation from monotherapy. Clinical trials lack head-to-head comparisons of second antihypertensive agents added to monotherapy, and only 2 trials (Avoiding Cardiovascular Events Through Combination Therapy in Patients Living With Systolic Hypertension [ACCOMPLISH]^5^ and Combination Therapy of Hypertension to Prevent Cardiovascular Events [COPE]^6^) directly compared combination regimens in patients with hypertension who required 2 drugs. These trials, however, provided comparisons between few agents, not drug classes; included primarily patients from Western countries; and did not systematically assess heterogeneity in patient subgroups. The absence of head-to-head comparison in clinical trials has limited the ability of clinical guidelines to provide recommendations about the preferred choice of second medication for treatment escalation.^7,8^ However, it is plausible that not all dual combination therapies have the same mean risks and benefits. Moreover, some combinations may be associated with better outcomes in particular patient subgroups. Evidence about the choice of medication in escalating treatment to 2 agents may better inform clinical decisions and provide needed evidence for practice guidelines.

To address these evidence gaps, we formed the Observational Health Data Science and Informatics (OHDSI) Asian Pacific collaboration group to conduct a series of large-scale observational studies that would investigate comparative effectiveness and safety associated with second antihypertensive agents added to monotherapy. This study is the first of the series of studies. Using data from 11 electronic health record (EHR) databases across 8 countries and regions, we aimed to investigate the most common dual combinations prescribed for treatment escalation in these countries and how treatment use varied by age, sex, and history of CVD. We hypothesized that there would be significant variation in use by country and patient subgroup. Specifically, we hypothesized that combinations with calcium channel blockers (CCBs) would be more commonly prescribed in Asian countries given prior reports on prescription patterns in these countries^9^ while combinations with angiotensin-converting enzyme inhibitors (ACEI) or angiotensin receptor blockers (ARBs) would be more commonly prescribed in Western countries, as recommended by current guidelines.^7,8^ Second, we hypothesized that certain combinations, such as ACEI or ARB and β-blocker, would be more commonly prescribed among patients with a history of CVD than those without a history of CVD. Secondary prevention guidelines recommend use of ACEIs in patients with prior myocardial infarction and use of β-blockers within 3 years of myocardial infarction (class I, level of evidence: A recommendation).^10^ We reported the treatment use of first-line antihypertensive monotherapies in a previous publication.^11^ In this study, we used a systematic, open-science, evidence-generation approach for high-quality observational research based on the Large-Scale Evidence Generation and Evaluation Across a Network of Databases for Hypertension (LEGEND-HTN) study.^11,12,13^ The results of this cohort study may provide insight into the current prescription patterns of dual combination therapies in hypertension treatment escalation and may lay a foundation for future work that compares the associations of different dual combinations with risk of cardiovascular outcomes.

## Methods

### Data Source

In this cohort study, we examined patient records from 11 EHR databases mapped to the Observational Medical Outcome Partnership (OMOP) Common Data Model (CDM) version 5.3 from participating research partners across the OHDSI community. These data sources included the Iqvia Longitudinal Patient Database (LPD) Australia (with 3 101 500 patients) and Electronic Practice-Based Research Network 2019 linked data set from South Western Sydney Local Health District (ePBRN SWSLHD; with 139 346 patients) from Australia, Ajou University School of Medicine (AUSOM; with 3 109 677 patients) and Kyung Hee University Hospital (KHMC; with 2 010 456 patients) databases from South Korea, Khoo Teck Puat Hospital (KTPH; with 290 074 patients) and National University Hospital (NUH; with 750 270 patients) databases from Singapore, Jiangsu Province Hospital (CJSPH; with 6 230 000 patients) database from China, Taipei Medical University Clinical Research Database (TMUCRD; with 3 659 572 patients) from Taiwan, Iqvia LPD France (with 18 118 000 patients) from France, Iqvia LPD Italy (with 2 209 600 patients) from Italy, and Iqvia US Ambulatory Electronic Medical Record (EMR; with 78 526 000 patients) database from the US (eAppendix 1 and eTable 1 in the Supplement). These data partners altogether monitor more than 118 million patients from 8 countries and regions across the world.

We executed this study through the federated network model of OHDSI, in which access to deidentified data and statistical analyses were executed inside each data partner’s institution using the OHDSI common tool stack.^11,12,13,14^ We prespecified the entire analytical process before execution and collected aggregated results from data partners for interpretation. Each data partner obtained the necessary institutional review board (IRB) approval or exemption and informed consent or exemption (eAppendix 2 in the Supplement). This study required no further ethics review or patient informed consent according to the policies of the Yale institutional review board. Previous studies have found that this process could be successfully applied to evaluate the comparative effectiveness of first-line antihypertensive monotherapies.^11,15,16^ This study followed the guidelines for cohort studies, described in the Strengthening the Reporting of Observational Studies in Epidemiology (STROBE) reporting guideline.

### Study Population

The study population consisted of adult patients (aged ≥18 years) with prior antihypertensive monotherapy who newly initiated escalated treatment with 1 of 56 drug ingredients (eTable 2 in the Supplement) that constitute 4 major drug classes, as recommended by current hypertension practice guidelines,^7,8,17,18^ from 2000 to 2019. Patients who initiated escalated treatment with other drug classes (eg, hydralazine or α-blockers) were excluded from analysis. For patients included in the analysis, we constructed 12 nonoverlapping exposure cohorts. Each cohort included patients who newly initiated 1 of 4 dual combination drug classes after escalating from monotherapy with 1 of 3 alternative classes (eTable 3 in the Supplement).

New-use cohort design is advocated as the primary design choice for comparative effectiveness research.^19^ The new-use design reduces confounding by identifying patients who start a new drug for treatment escalation, using initiation of the second drug as the start of follow-up.

Specifically, cohort entry (ie, index date) for each patient was the patient’s date of prescription initiating the second drug containing the RxNorm ingredient codes of 1 of the 4 major drug classes from 2000 to 2019. Inclusion criteria for patients based on index date included at least 1 hypertension diagnosis any time in the patient’s record before the index date, at least 1 year of observation time before the index date (ie, a washout period to improve new-use sensitivity), at least 1 prescription of an antihypertensive agent and no prescriptions for other agents any time before the index date, and at least 30 days between initiation of first drug class and initiation of second drug class on the index date (eFigure 1 in the Supplement). This analysis focused on the second agent added after antihypertensive monotherapy rather than on switching of medications. We purposefully did not exclude patients with a history of CVD events, enabling us to report drug use for individuals with and without history of CVD. History of CVD was defined as at least 1 diagnosis code for arteriosclerotic vascular disease, cerebrovascular disease, ischemic heart disease, or peripheral vascular disease any time on or prior to the index date. Continuous drug exposures were constructed by allowing smaller than 30-day gaps between prescriptions.

### Cohort Development and Validation

We developed exposure cohorts previously listed using OHDSI’s open-source Atlas^20^ platform that enables researchers to define cohorts based on drug exposures, diagnoses, procedures, and patient characteristics through a user-friendly interface. We based drug exposure on occurrences of RxNorm codes in the appropriate OMOP CDM tables and built diagnosis concept sets, such as hypertension diagnosis, as Systemized Nomenclature of Medicine-Clinical Terms term collections in appropriate OMOP CDM tables. Atlas enforced complete transparency in cohort definitions by automatically generating human-readable and computer-readable representations. We used previously validated concept definitions for hypertension diagnosis and antihypertensive agents.^11^

We further validated exposure cohorts and aggregated drug use against data sources using comprehensive cohort characterization tools through OHDSI’s CohortDiagnostic package.^21^ For each cohort and data source, this package systematically generated incidence new-use rates (stratified by age, sex, and calendar year), cohort characteristics (ie, all comorbidities, drug use, and health care use), and codes found in patient records that triggered various rules in cohort definitions. This approach allowed us to better understand the heterogeneity of source coding for exposures and health outcomes, as well as the association of various inclusion criteria with overall cohort counts.

### Statistical Analysis

For each database, we described overall use in dual combination therapies and evaluated treatment variation in patient groups by age (ie, ages 18-64 and ≥65 years), sex, history of CVD, and country. Specifically, we calculated the proportion of patients treated with each dual combination regimen. We compared the distribution of treatment use across countries and between patient subgroups defined by age, sex, and history of CVD using χ^2^ tests. A prespecified 2-sided *P* value < .05 was used as the level of statistical significance. In addition to the use of *P* values, we conducted meta-analyses to quantify between-country heterogeneity and used *I^2^* to describe the percentage of variability in estimates associated with between-country heterogeneity rather than sampling variations.^22^ Finally, we characterized treatment pathways for hypertension (ie, the ordered sequence of medications that a patient was prescribed) in diverse populations using sunburst plots. Sequences included changes in medication and additions of medication. All analyses were performed using R statistical software version 4.0 (R Project for Statistical Computing).

## Results

### Use of Dual Combination Therapies in Treatment Escalation

Across 11 data sources, our final analysis included 970 335 patients with hypertension who newly initiated dual combinations of antihypertensive agents after escalating from monotherapy: 11 494 patients from Australia (including 9291 patients in Australia LPD and 2203 patients in ePBRN SWSLHD), 6980 patients from South Korea (including 6029 patients in Ajou University and 951 patients in KHMC), 2096 patients from Singapore (including 842 patients from KTPH and 1254 patients from NUH), 7008 patients from China, 8544 patients from Taiwan, 103 994 patients from France, 76 082 patients from Italy, and 754 137 patients from the US (Table 1). Patient mean (SD) age varied across data sources, ranging from 57.6 (14.8) years in China to 67.7 (15.9) years in Singapore’s KTPH database. The proportion of patients by sex ranged from 24 358 (36.9%) women in Italy to 408 964 (54.3%) women in the US, and the proportion of patients with a history of CVD ranged from 1350 patients in Australia’s ePBRN SWSLHD (10.6%) to 536 patients (56.4%) in Korea’s KHMC database.

**Table 1.** Use of 12 Dual Antihypertensive Medication Combinations From 11 Committed Data Sources

Dual combination^a^	Patients in data source, No. (%) (N = 970 335)										
	Australia		South Korea		Singapore		China	Taiwan	France	Italy	US
	Australia LPD (n = 9291)	ePBRN SWSLHD (n = 2203)	Ajou University (n = 6029)	KHMC (n = 951)	KTPH (n = 842)	NUH (n = 1254)	Jiangsu (n = 7008)	TMUCRD (n = 8544)	France LPD (n = 103 994)	Italy LPD (n = 76 082)	US AmbEMR (n = 754 137)
Starting with ACEI or ARB	6762 (72.8)	1474 (66.9)	2082 (34.5)	208 (21.9)	337 (40)	614 (49)	3284 (46.9)	2296 (26.9)	56 158 (54)	43460 (57.1)	32 9803 (43.7)
+CCB	3842 (41.4)	698 (31.7)	1216 (20.2)	147 (15.5)	216 (25.7)	439 (35)	3127 (44.6)	1545 (18.1)	22 523 (21.7)	14268 (19.2)	95 248 (12.6)
+β-blocker	1078 (11.6)	268 (12.2)	392 (6.5)	49 (5.2)	105 (12.5)	144 (11.5)	46 (0.7)	748 (8.8)	11 236 (10.8)	11844 (15.6)	11 0556 (14.7)
+Diuretic	1842 (19.8)	508 (23.1)	474 (7.9)	12 (1.3)	16 (1.9)	31 (2.5)	111 (1.6)	3 (0)	22 399 (21.5)	16988 (22.3)	12 3940 (16.4)
Starting with CCB	1454 (15.7)	315 (14.3)	2560 (42.5)	423 (44.5)	322 (38.2)	240 (19.1)	3424 (48.9)	3834 (44.9)	21 275 (20.5)	9419 (12.4)	10 5998 (14.1)
+ACEI or ARB	1212 (13.0)	246 (11.2)	1487 (24.7)	191 (20.1)	191 (22.7)	133 (10.6)	3312 (47.3)	2651 (31)	15 749 (15.1)	5841 (7.7)	54 297 (7.2)
+β-blocker	178 (1.9)	41 (1.9)	814 (13.5)	217 (22.8)	120 (14.3)	101 (8.1)	34 (0.5)	1182 (13.8)	3866 (3.7)	2475 (3.3)	30 593 (4.1)
+Diuretic	64 (0.7)	28 (1.3)	259 (4.3)	15 (1.6)	11 (1.3)	6 (0.5)	78 (1.1)	1 (0)	1660 (1.6)	1103 (1.5)	21 108 (2.8)
Starting with β-blocker	806 (8.7)	281 (12.8)	1051 (17.4)	307 (32.3)	170 (20.2)	378 (30.2)	46 (0.7)	2414 (28.3)	21 404 (20.6)	13986 (18.4)	18 4071 (24.4)
+ACEI or ARB	635 (6.8)	210 (9.5)	386 (6.4)	98 (10.3)	68 (8.1)	128 (10.2)	26 (0.4)	1250 (14.6)	11 116 (10.7)	8264 (10.9)	10 6380 (14.1)
+CCB	145 (1.6)	54 (2.5)	614 (10.2)	199 (20.9)	97 (11.5)	243 (19.4)	19 (0.3)	1163 (13.6)	5972 (5.7)	2755 (3.6)	41 388 (5.5)
+Diuretic	26 (0.3)	17 (0.8)	51 (0.9)	10 (1.1)	5 (0.6)	7 (0.6)	1 (0)	1 (0)	4316 (4.2)	2967 (3.9)	36 303 (4.8)
Starting with diuretic	269 (2.9)	133 (6)	336 (5.6)	13 (1.4)	13 (1.6)	22 (1.8)	254 (3.6)	0	5157 (5)	9217 (12.1)	13 4265 (17.8)
+ACEI or ARB	206 (2.2)	94 (4.3)	154 (2.6)	2 (0.2)	8 (1)	7 (0.6)	114 (1.6)	0	3281 (3.2)	5749 (7.6)	84 275 (11.2)
+CCB	42 (0.5)	25 (1.1)	139 (2.3)	6 (0.6)	4 (0.5)	7 (0.6)	140 (2.0)	0	1097 (1.1)	1539 (2.0)	22 568 (3.0)
+β-blocker	21 (0.2)	14 (0.6)	43 (0.7)	5 (0.5)	1 (0.1)	8 (0.6)	0	0	779 (0.8)	1929 (2.5)	27 422 (3.6)

Abbreviations: ACEI, angiotensin converting enzyme inhibitor; AmbEMR, Ambulatory Electronic Medical Record; ARB, angiotensin receptor blocker; CCB, calcium channel blocker; ePBRN SWSLHD, Electronic Practice-based Research Network 2019 linked data set from South Western Sydney Local Health District; KHMC, Kyung Hee University Hospital; KTPH, Khoo Teck Puat Hospital; LPD, Longitudinal Patient Database; NUH, National University Hospital; TMUCRD, Taiwan Taipei Medical University Clinical Research Database.

^a^ Treatments linked with a + indicate a monotherapy followed by a second therapy. For example, ACEI or ARB + β-blocker indicates starting an ACEI or ARB monotherapy followed by a β-blocker.

In the overall cohort, the most commonly prescribed combination was ACEI or ARB + thiazide or thiazide-like diuretic (hereafter, *diuretic*; 166 324 patients [17.1%]). Table 1 reports the treatment use across countries. Starting an ACEI or ARB monotherapy followed by a CCB (ie, ACEI or ARB + CCB) was the most commonly prescribed combination in Australia (698 patients in ePBRN SWSLHD [31.7%] and 3842 patients in Australia LPD [41.4%]) and Singapore (216 patients in KTPH [25.7%] and 439 patients in NUH [35.0%]). ACEI or ARB + diuretic was the most commonly prescribed combination in Italy (16 988 patients [22.3%]) and the US (123 940 patients [16.4%]). In South Korea, CCB + ACEI or ARB (191 patients in KHMC [20.1%] and 1487 patients in Ajou University [24.7%]), CCB + β-blocker (814 patients in Ajou University [13.5%] and 217 patients in KHMC [22.8%]), and ACEI or ARB + CCB (147 patients in KHMC [15.5%] and 1216 patients in Ajou University [20.2%]) were the 3 commonly prescribed combinations. In China, CCB + ACEI or ARB (3312 patients [47.3%]) and ACEI or ARB + CCB (3127 patients [44.6%]) were the 2 most commonly prescribed combinations, whereas in France, ACEI or ARB + CCB (22 523 patients [21.7%]) and ACEI or ARB + diuretic (22 399 patients [21.5%]) were the 2 most commonly prescribed combinations. The treatment pattern was statistically significantly different across countries (eFigure 2 in the Supplement).

In Western countries (ie, Australia, France, Italy, and the US), the proportion of patients treated with ACEI or ARB + diuretic ranged from 123 940 patients (16.4%) in the US to 508 patients (23.1%) in Australia’s ePBRN SWSLHD, whereas the proportion in Asian countries (ie, South Korea, Singapore, China, and Taiwan) ranged from 3 patients in Taiwan (0.04%) to 474 patients in South Korea’s Ajou University (7.9%). Among Western countries, the proportion of patients treated with CCB + ACEI or ARB ranged from 54 297 patients in the US (7.2%) to 15 749 patients in France (15.1%), whereas the proportion among Asian countries ranged from 133 patients in Singapore’s NUH (10.6%) to 3312 patients in China (47.3%). Forest plots for treatment proportions for each dual combination across different countries are presented in eFigure 2 in the Supplement. Almost all observed differences in treatment proportions were associated with between-country heterogeneity, and sampling variations were associated with a negligible part (<0.4%) of observed variations.

Table 2 shows treatment use by age group. In each database, the most commonly prescribed combinations for patients ages 18 to 64 years and ages 65 years or older was generally similar. For example, in Australian databases, there were 1232 patients aged 18 to 64 years and 971 patients aged 65 years or older in ePBRN SWSLHD and 5248 patients aged 18 to 64 years and 4044 patients aged 65 years or older in Australia LPD, and the most commonly prescribed combination was ACEI or ARB + CCB for patients aged 18 to 64 years and those ages 65 years and older (ePBRN SWSLHD: 393 patients [31.9%] and 305 patients [31.4%]; Australia LPD: 2433 patients [46.4%] and 1409 patients [34.9%]). In the Italy LPD database, the most commonly prescribed combination was ACEI or ARB + diuretic among 34 209 patients ages 18 to 64 years (8096 patients [23.7%]) and 41 873 patients ages 65 years (8892 patients [21.2%]). However, some combination therapies had wide variation of usage across age groups. For example, in the Australian databases, the proportions of patients treated with ACEI or ARB + β-blocker was 417 patients ages 18 to 64 years (8.0%) and 661 patients ages 65 years and older (16.4%) in Australia LPD and 121 patients ages 18 to 64 years (9.9%) and 147 patients ages 65 years and older (15.1%) in ePBRN SWSLHD. Use of ACEI or ARB + CCB varied from 873 of 3737 patients ages 18 to 64 years (23.4%) to 343 of 2292 patients ages 65 years or older (15.0%) in South Korea’s Ajou University database. The distribution of all 12 dual combination therapies were significantly different by age in all databases (*P* for drug combination distributions by age < .001), except for the South Korean KHMC database.

**Table 2.** Use of 12 Dual Antihypertensive Medication Combinations by Age

Dual combination therapy^a^	Patients, No. (%)																					
	Australia				South Korea				Singapore NUH		**China Jiangsu**		**Taiwan TMUCRD**		**France LPD**			**Italy LPD**			US AmbEMR	
	Australia LPD		ePBRN SWSLHD		Ajou University		KHMC															
	Ages 18-64 y	Ages ≥65 y	Ages 18-64 y	Ages ≥65 y	Ages 18-64 y	Ages ≥65 y	Ages 18-64 y	Ages ≥65 y	Ages 18-64 y	Ages ≥65 y	Ages 18-64 y	Ages ≥65 y	Ages 18-64 y	Ages ≥65 y	Ages 18-64 y		Ages ≥65 y	Ages 18-64 y		Ages ≥65 y	Ages 18-64 y	Ages ≥65 y
Total	5248	4044	1232	971	3737	2292	399	552	482	773	4656	2352	5226	3318	49 727	54 269		34 209	41 873		391 018	363 119
ACEI or ARB																						
+CCB	2433 (46.4)	1409 (34.9)	393 (31.9)	305 (31.4)	873 (23.4)	343 (15)	58 (14.5)	89 (16.2)	159 (33.1)	280 (36.2)	2057 (44.2)	1070 (45.5)	874 (16.7)	671 (20.2)	11132 (22.4)	11391 (21)		6496 (19)	8132 (19.4)		43211 (11.1)	52 073 (14.3)
+β-blocker	417 (8.0)	661 (16.4)	121 (9.9)	147 (15.1)	241 (6.4)	151 (6.6)	20 (5)	29 (5.3)	61 (12.7)	83 (10.7)	31 (0.7)	15 (0.6)	482 (9.2)	266 (8)	4647 (9.3)	6589 (12.1)		4406 (12.9)	7438 (17.8)		56003 (14.3)	54 576 (15)
+Diuretic	1132 (21.6)	710 (17.6)	340 (27.6)	168 (17.3)	328 (8.8)	146 (6.4)	5 (1.3)	7 (1.3)	17 (3.5)	14 (1.8)	74 (1.6)	37 (1.6)	1 (0)	2 (0.1)	11384 (22.9)	11015 (20.3)		8096 (23.7)	8892 (21.2)		70856 (18.1)	53 084 (14.6)
CCB																						
+ACEI or ARB	615 (11.7)	597 (14.8)	115 (9.4)	131 (13.4)	906 (24.2)	581 (25.4)	79 (19.8)	112 (20.3)	51 (10.7)	82 (10.6)	2204 (47.3)	1108 (47.1)	1560 (29.9)	1091 (32.9)	7879 (15.8)	7871 (14.5)		2447 (7.2)	3395 (8.1)		24417 (6.2)	29 880 (8.2)
+β-blocker	74 (1.4)	104 (2.6)	15 (1.2)	26 (2.7)	414 (11.1)	400 (17.5)	88 (22.0)	129 (23.4)	49 (10.3)	52 (6.7)	26 (0.6)	8 (0.3)	700 (13.4)	482 (14.5)	1624 (3.3)	2242 (4.1)		908 (2.7)	1567 (3.7)		13938 (3.6)	16 655 (4.6)
+Diuretic	28 (0.5)	36 (0.9)	13 (1.1)	15 (1.5)	134 (3.6)	125 (5.5)	6 (1.5)	9 (1.6)	3 (0.6)	3 (0.4)	56 (1.2)	22 (0.9)	0	1 (0)	726 (1.5)	935 (1.7)		424 (1.2)	678 (1.6)		11263 (2.9)	9845 (2.7)
β-blocker																						
+ACEI or ARB	359 (6.8)	276 (6.8)	111 (9.0)	99 (10.2)	256 (6.8)	130 (5.7)	50 (12.5)	48 (8.7)	57 (11.8)	71 (9.2)	19 (0.4)	7 (0.3)	851 (16.3)	399 (12)	5277 (10.6)	5839 (10.8)		4383 (12.8)	3881 (9.3)		54413 (13.9)	51 967 (14.3)
+CCB	67 (1.3)	78 (1.9)	30 (2.4)	24 (2.5)	328 (8.8)	286 (12.5)	86 (21.5)	113 (20.5)	71 (14.8)	172 (22.2)	12 (0.3)	7 (0.3)	758 (14.5)	405 (12.2)	2506 (5)	3466 (6.4)		1421 (4.2)	1334 (3.2)		19171 (4.9)	22 217 (6.1)
+Diuretic	11 (0.2)	15 (0.4)	8 (0.6)	9 (0.9)	29 (0.8)	22 (1.0)	3 (0.8)	7 (1.3)	4 (0.7)	2 (0.3)	1 (0)	0	0	1 (0)	2286 (4.6)	2030 (3.7)		1786 (5.2)	1181 (2.8)		20348 (5.2)	15 955 (4.4)
Diuretic																						
+ACEI or ARB	15 (0.3)	27 (0.7)	12 (1.0)	13 (1.3)	92 (2.5)	47 (2.1)	2 (0.5)	4 (0.7)	1 (0.2)	6 (0.8)	78 (1.7)	36 (1.5)	0	0	1534 (3.1)	1747 (3.2)		2442 (7.1)	3307 (7.9)		49689 (12.7)	34 586 (9.5)
+CCB	90 (1.7)	116 (2.9)	65 (5.3)	29 (3.0)	108 (2.9)	46 (2)	1 (0.3)	1 (0.2)	5 (1.0)	4 (0.6)	99 (2.1)	41 (1.7)	0	0	413 (0.8)	685 (1.3)		552 (1.6)	987 (2.4)		12357 (3.2)	10 211 (2.8)
+β-blocker	9 (0.2)	12 (0.3)	8 (0.6)	6 (0.6)	30 (0.8)	13 (0.6)	2 (0.5)	3 (0.5)	3 (0.6)	5 (0.6)	0	0	0	0	320 (0.6)	459 (0.8)		846 (2.5)	1083 (2.6)		15351 (3.9)	12 071 (3.3)

Abbreviations: ACEI, angiotensin-converting enzyme inhibitor; AmbEMR, Ambulatory Electronic Medical Record; ARB, angiotensin receptor blocker; CCB, calcium channel blocker; ePBRN SWSLHD, Electronic Practice-based Research Network 2019 linked data set from South Western Sydney Local Health District; KHMC, Kyung Hee University Hospital; LPD, Longitudinal Patient Database; NUH, National University Hospital; TMUCRD, Taiwan Taipei Medical University Clinical Research Database.

^a^ Treatments linked with a + indicate a monotherapy followed by a second therapy. For example, ACEI or ARB + β-blocker indicates starting an ACEI or ARB monotherapy followed by a β-blocker.

Table 3 shows treatment use by sex. In each database, the 3 most commonly prescribed combinations in men and women were generally similar. For example, in the Australian databases, the 3 most commonly prescribed combinations among 1054 men and 1149 women in ePBRN SWSLHD and 4718 men and 4549 women in Australia LPD were ACEI or ARB + CCB (ePBRN SWSLHD: 359 [34.1%] men and 339 [29.5%] women; Australia LPD: 2121 [44.8%] men and 1721 [37.7%] women), ACEI or ARB + diuretic (Australia LPD: 870 [18.4%] men and 972 [21.3%] women; ePBRN SWSLHD: 247 [23.4%] men and 261 [22.7%] women), and ACEI or ARB + β-blocker (ePBRN SWSLHD:129 [12.2%] men and 490 [10.7%] women; Australia LPD: 589 [12.4%] men and 139 [12.1%] women). In the Italy database, the 3 most commonly prescribed combinations among 46 656 men and 29 427 women were ACEI or ARB + diuretic (10 242 [22.0%] men and 6746 [22.6%] women), ACEI or ARB + CCB (7653 [16.4%] men and 6975 [23.7%] women), and ACEI or ARB + β-blocker (7093 [15.2%] men and 4752 [16.1%] women). However, the distribution of all 12 dual combination therapies were significantly different by sex in all databases. For example, use of ACEI or ARB + CCB varied from 2121 of 4718 (44.8%) men to 1721 of 4549 (37.7%) women in Australian LPD (*P* for drug combination distributions by sex < .001).

**Table 3.** Use of 12 Dual Antihypertensive Medication Combinations by Sex

Dual combination therapy^a^	Patients, No. (%)																			
	Australia				South Korea				Singapore NUH		**China Jiangsu**		**Taiwan TMUCRD**		**France LPD**		**Italy LPD**		US AmbEMR	
	Australia LPD		ePBRN SWSLHD		Ajou University		KHMC													
	Men	Women	Men	Women	Men	Women	Men	Women	Men	Women	Men	Women	Men	Women	Men	Women	Men	Women	Men	Women
Total	4718	4549	1054	1149	3068	2961	449	502	656	598	3777	3231	4100	4444	48 843	55 151	46 656	29 427	344 942	409 195
ACEI or ARB																				
+CCB	2121 (44.8)	1721 (37.7)	359 (34.1)	339 (29.5)	695 (22.7)	521 (17.6)	70 (15.6)	77 (15.3)	205 (31.2)	234 (39.1)	1707 (45.2)	1420 (43.9)	708 (17.3)	837 (18.8)	12072 (24.7)	10452 (19)	7653 (16.4)	6975 (23.7)	51 221 (14.8)	44 063 (10.8)
+β-blocker	589 (12.4)	490 (10.7)	129 (12.2)	139 (12.1)	213 (6.9)	179 (6.0)	30 (6.7)	19 (3.8)	90 (13.7)	54 (9)	22 (0.6)	24 (0.7)	340 (8.3)	408 (9.2)	5578 (11.4)	5658 (10.3)	7093 (15.2)	4752 (16.1)	60 128 (17.4)	50 451 (12.3)
+Diuretic	870 (18.4)	972 (21.3)	247 (23.4)	261 (22.7)	261 (8.5)	213 (7.2)	3 (0.7)	9 (1.8)	12 (1.8)	19 (3.2)	62 (1.6)	49 (1.5)	0	3 (0.1)	10879 (22.3)	11520 (20.9)	10242 (22.0)	6746 (22.9)	60 551 (17.6)	63 389 (15.5)
CCB																				
+ACEI or ARB	617 (13.1)	595 (13)	128 (12.1)	118 (10.3)	791 (25.8)	696 (23.5)	89 (19.8)	102 (20.3)	58 (8.8)	75 (12.5)	1765 (46.7)	1547 (47.9)	1175 (28.7)	1476 (33.2)	8052 (16.5)	7696 (14.0)	3173 (6.8)	2668 (9.1)	26 498 (7.7)	27 800 (6.8)
+β-blocker	80 (1.7)	98 (2.2)	18 (1.7)	23 (2.0)	392 (12.8)	422 (14.3)	99 (22)	118 (23.5)	63 (9.6)	38 (6.4)	17 (0.5)	17 (0.5)	608 (14.8)	574 (12.9)	1592 (3.3)	2274 (4.1)	1515 (3.2)	960 (3.3)	13 173 (3.8)	17 419 (4.3)
+Diuretic	22 (0.5)	42 (0.9)	15 (1.4)	13 (1.1)	105 (3.4)	154 (5.2)	2 (0.4)	13 (2.6)	4 (0.6)	2 (0.3)	40 (1.1)	38 (1.2)	1 (0)	0	679 (1.4)	981 (1.8)	706 (1.5)	397 (1.3)	7568 (2.2)	13 540 (3.3)
β-blocker																				
+ACEI or ARB	292 (6.2)	343 (7.5)	82 (7.8)	128 (11.1)	211 (6.9)	175 (5.9)	58 (12.9)	40 (8)	81 (12.3)	47 (7.9)	15 (0.4)	11 (0.3)	632 (15.4)	618 (13.9)	4586 (9.4)	6531 (11.8)	5410 (11.6)	2854 (9.7)	53 976 (15.6)	52 404 (12.8)
+CCB	58 (1.2)	87 (1.9)	23 (2.2)	31 (2.7)	290 (9.5)	324 (10.9)	88 (19.6)	111 (22.1)	129 (19.7)	114 (19.1)	11 (0.3)	8 (0.2)	635 (15.5)	528 (11.9)	2167 (4.4)	3805 (6.9)	1825 (3.9)	930 (3.2)	17 796 (5.2)	23 592 (5.8)
+Diuretic	7 (0.2)	19 (0.4)	7 (0.7)	10 (0.9)	14 (0.5)	37 (1.2)	4 (0.9)	6 (1.2)	3 (0.5)	4 (0.7)	1 (0)	0	1 (0)	0	1342 (2.7)	2926 (5.3)	2275 (4.9)	692 (2.4)	11 932 (3.5)	24 371 (6.0)
Diuretic																				
+ACEI or ARB	53 (1.1)	153 (3.4)	32 (3)	62 (5.4)	41 (1.3)	113 (3.8)	1 (0.2)	1 (0.2)	3 (0.5)	4 (0.7)	58 (1.5)	56 (1.7)	0	0	1208 (2.5)	2073 (3.8)	4212 (9)	1537 (5.2)	28 067 (8.1)	56 208 (13.7)
+CCB	16 (0.3)	26 (0.6)	9 (0.9)	16 (1.4)	43 (1.4)	96 (3.2)	4 (0.9)	2 (0.4)	3 (0.5)	4 (0.7)	79 (2.1)	61 (1.9)	0	0	414 (0.8)	683 (1.2)	1068 (2.3)	471 (1.6)	6771 (2.0)	15 796 (3.9)
+β-blocker	4 (0.1)	17 (0.4)	5 (0.5)	9 (0.8)	12 (0.4)	31 (1)	1 (0.2)	4 (0.8)	5 (0.8)	3 (0.5)	0	0	0	0	251 (0.5)	528 (1)	1484 (3.2)	445 (1.5)	7260 (2.1)	20 162 (4.9)

Abbreviations: ACEI, angiotensin-converting enzyme inhibitor; AmbEMR, Ambulatory Electronic Medical Record; ARB, angiotensin receptor blocker; CCB, calcium channel blocker; ePBRN SWSLHD, Electronic Practice-based Research Network 2019 linked data set from South Western Sydney Local Health District; KHMC, Kyung Hee University Hospital; LPD, Longitudinal Patient Database; NUH, National University Hospital; TMUCRD, Taiwan Taipei Medical University Clinical Research Database.

^a^ Treatments linked with a + indicate a monotherapy followed by a second therapy. For example, ACEI or ARB + β-blocker indicates starting an ACEI or ARB monotherapy followed by a β-blocker.

Table 4 shows treatment use by history of CVD. Treatment patterns of dual combination therapies were significantly different by history of CVD in all databases (*P* for drug combination distributions by CVD < .001). In all databases except the China Jiangsu database, there was wide variation in use of ACEI or ARB + β-blocker by history of CVD. For example, in the US database, the proportion of patients receiving ACEI or ARB + β-blocker was 37 663 of 169 687 patients with a history of CVD (22.2%) and 72 916 of 584 450 patients without a history of CVD (12.5%). Similarly, in all databases except the Australian ePBRN SWSLHD and China Jiangsu databases, there was wide variation in use of β-blocker + ACEI or ARB by history of CVD. For example, in the US database, the proportion of patients receiving β-blocker + ACEI or ARB was 37 882 patients (22.3%) with a history of CVD and 68 498 patients (11.7%) without a history of CVD.

**Table 4.** Use of 12 Dual Antihypertensive Medication Combinations by History of CVD

Dual combination therapy^a^	Patients, No. (%)																			
	Australia				South Korea				Singapore NUH		**China Jiangsu**		**Taiwan TMUCRD**		**France LPD**		**Italy LPD**		**US AmbEMR**	
	Australia LPD		ePBRN SWSLHD		Ajou University		KHMC													
	With CVD	Without CVD	With CVD	Without CVD	With CVD	Without CVD	With CVD	Without CVD	With CVD	Without CVD	With CVD	Without CVD	With CVD	Without CVD	With CVD	Without CVD	With CVD	Without CVD	With CVD	Without CVD
Total	1350	7941	233	1970	1521	4508	536	415	446	808	2170	4838	2774	5770	14 304	89 690	14 791	61 291	169 687	584 450
ACEI or ARB																				
+CCB	447 (33.1)	3395 (42.7)	68 (29.2)	630 (32.0)	307 (20.2)	909 (20.2)	97 (18.1)	50 (12.0)	131 (29.4)	308 (38.1)	897 (41.3)	2230 (46.1)	465 (16.8)	1080 (18.7)	2923 (20.4)	19600 (21.9)	2838 (19.2)	11790 (19.2)	18218 (10.7)	77066 (13.2)
+β-blocker	333 (24.7)	745 (9.4)	52 (22.3)	216 (11.0)	129 (8.5)	263 (5.8)	38 (7.1)	11 (2.7)	57 (12.8)	87 (10.8)	11 (0.5)	35 (0.7)	267 (9.6)	481 (8.3)	2412 (16.9)	8824 (9.8)	3154 (21.3)	8690 (14.2)	37663 (22.2)	72916 (12.5)
+Diuretic	153 (11.4)	1689 (21.3)	30 (12.9)	478 (24.3)	75 (4.9)	399 (8.9)	5 (0.9)	7 (1.7)	8 (1.8)	23 (2.8)	27 (1.2)	84 (1.7)	3 (0.1)	0	2141 (15)	20258 (22.6)	2557 (17.3)	14431 (23.5)	14811 (8.7)	109129 (18.7)
CCB																				
+ACEI or ARB	176 (13)	1036 (13.1)	32 (13.7)	214 (10.9)	383 (25.2)	1104 (24.5)	109 (20.3)	82 (19.8)	30 (6.7)	103 (12.7)	1140 (52.5)	2172 (44.9)	750 (27)	1901 (32.9)	2091 (14.6)	13658 (15.2)	1296 (8.8)	4545 (7.4)	10468 (6.2)	43829 (7.5)
+β-blocker	14 (1.1)	164 (2.1)	9 (3.9)	32 (1.6)	242 (15.9)	572 (12.7)	98 (18.3)	119 (28.7)	36 (8.1)	65 (8)	5 (0.2)	29 (0.6)	399 (14.4)	783 (13.6)	679 (4.7)	3187 (3.6)	630 (4.3)	1845 (3.0)	9618 (5.7)	20975 (3.6)
+Diuretic	10 (0.8)	54 (0.7)	1 (0.4)	27 (1.4)	46 (3)	213 (4.7)	6 (1.1)	9 (2.2)	1 (0.2)	5 (0.6)	20 (0.9)	58 (1.2)	1 (0)	0	237 (1.7)	1423 (1.6)	177 (1.2)	926 (1.5)	3268 (1.9)	17840 (3.1)
β-blocker																				
+ACEI or ARB	143 (10.6)	492 (6.2)	21 (9.0)	189 (9.6)	114 (7.5)	272 (6)	68 (12.7)	30 (7.2)	72 (16.1)	56 (6.9)	2 (0.1)	24 (0.5)	475 (17.1)	775 (13.4)	1787 (12.5)	9329 (10.4)	1735 (11.7)	6529 (10.7)	37882 (22.3)	68498 (11.7)
+CCB	27 (2.0)	118 (1.5)	8 (3.4)	46 (2.3)	165 (10.8)	449 (10.0)	105 (19.6)	94 (22.7)	101 (22.6)	142 (17.6)	9 (0.4)	10 (0.2)	414 (14.9)	749 (13)	981 (6.9)	4991 (5.6)	521 (3.5)	2234 (3.6)	13981 (8.2)	27407 (4.7)
+Diuretic	4 (0.3)	22 (0.3)	3 (1.3)	14 (0.7)	11 (0.7)	40 (0.9)	1 (0.2)	4 (1.0)	3 (0.7)	4 (0.5)	1 (0)	0	0	1 (0)	424 (3.0)	3892 (4.3)	301 (2)	2666 (4.4)	7504 (4.4)	28799 (4.9)
Diuretic																				
+ACEI or ARB	23 (1.7)	183 (2.3)	8 (3.4)	86 (4.4)	23 (1.5)	131 (2.9)	1 (0.2)	1 (0.2)	2 (0.4)	5 (0.6)	25 (1.2)	89 (1.8)	0	0	355 (2.5)	2926 (3.3)	953 (6.4)	4796 (7.8)	8300 (4.9)	75975 (13)
+CCB	11 (0.8)	31 (0.4)	1 (0.4)	24 (1.2)	20 (1.3)	119 (2.6)	3 (0.6)	3 (0.7)	1 (0.2)	6 (0.7)	33 (1.5)	107 (2.2)	0	0	156 (1.1)	941 (1.0)	292 (2.0)	1247 (2.0)	3038 (1.8)	19530 (3.3)
+β-blocker	6 (0.5)	15 (0.2)	0	14 (0.7)	6 (0.4)	37 (0.8)	1 (0.2)	4 (1)	4 (0.9)	4 (0.5)	0	0	0	0	116 (0.8)	663 (0.7)	338 (2.3)	1591 (2.6)	4936 (2.9)	22486 (3.8)

Abbreviations: ACEI, angiotensin-converting enzyme inhibitor; AmbEMR, Ambulatory Electronic Medical Record; ARB, angiotensin receptor blocker; CCB, calcium channel blocker; CVD, cardiovascular disease; ePBRN SWSLHD, Electronic Practice-based Research Network 2019 Linked Data set from South Western Sydney Local Health District; KHMC, Kyung Hee University Hospital; LPD, Longitudinal Patient Database; NUH, National University Hospital; TMUCRD, Taiwan Taipei Medical University Clinical Research Database.

^a^ Treatments linked with a + indicate a monotherapy followed by a second therapy. For example, ACEI or ARB + β-blocker indicates starting an ACEI or ARB monotherapy followed by a β-blocker.

### Treatment Pathway for Hypertension

Tracking medication changes for these 970 335 patients over time revealed a diverse array of treatment trajectories across countries. Treatment pathways for hypertensive agents across the largest 9 data sources are shown in eFigure 3 in the Supplement. The most common first-line therapy among patients in Australia and Singapore was an ACEI or ARB, whereas the most common first-line therapy among patients in South Korea was a CCB. The proportion of patients who were prescribed dual therapy differed among countries. There were more patients in Australia who initiated dual therapy than in South Korea. Most patients (5893 patients [84.1%]) in China initiated with a CCB or an ACEI or ARB. The common prescription in the US, Italy, and France was an ACEI or ARB and a diuretic.

## Discussion

In this cohort study, we observed heterogeneity in the use of dual combination therapies as recorded in EHR data sources, identifying a total of 970 335 patients with hypertension and dual combination therapy in Australia, South Korea, Singapore, China, Taiwan, France, Italy, and the US. To our knowledge, this is the first study to describe use in clinical practice of antihypertensive dual combination therapies for treatment escalation across 8 countries, including 5 Asia Pacific countries and regions. These findings may provide insight into the current prescription patterns of antihypertensive agents and lay a foundation for future studies to investigate the comparative effectiveness associated with different antihypertensive combinations. Such information is important to inform clinical decisions given the large number of patients who require combination therapy and the lack of evidence on which combinations are associated with the best balance in risks and benefits.

Our study extends prior literature given that, to our knowledge, it is the largest multisite analysis of evidence from clinical practice to address dual combination therapies used in treatment escalation of hypertension. Through the OHDSI community, particularly the OHDSI Asia-Pacific (APAC network), we took advantage of disparate health databases drawn from different sources and across a range of countries and practice settings. These large-scale and unfiltered populations represent clinical practice data. This first descriptive analysis of the OHDSI APAC collaborative suggests that coordinated efforts may be able to overcome many logistic and methodological challenges associated with observational study designs. The profiles of treatment pathways are based on more than 118 million patient records. We successfully addressed patient privacy and diverse research regulatory constraints, adopted a consistent data model, and distributed queries across a broad population.

There are several possible explanations for our findings. The observed prescription pattern of antihypertensive agents is, in part, a reflection of hypertension guidelines issued in the past few decades. An ACEI or ARB was the most commonly prescribed drug class across all data sources, which may be expected given that it is recommended as a first-line treatment option by most guidelines.^8,17,18,23,24,25^ The finding that CCBs were the predominant prescribed drug class in the Chinese data source is consistent with a previous national study in China,^9^ which may reflect the endorsement of the clinical guideline in China and the lower cost of CCBs compared with other antihypertensive drugs.^18^ Despite findings suggesting that β-blockers are less effective for stroke,^26,27^ their high use rates in South Korea and the US are consistent with nationwide studies^11,17,28^ in those countries finding that use of β-blocker monotherapy for patients with hypertension remains prevalent. Among patients with a history of CVD, the common use of an ACEI or ARB or a β-blocker is consistent with guidelines for secondary prevention of CVD.^10,29,30^ Additionally, our study corroborates previous work by OHDSI researchers, which found significant heterogeneity in treatment pathways for several chronic diseases across data sources.^11,15,31^ Variation in treatment use could also be associated with difference in the selection of patients in each data source, particularly owing to the difference in proportion of patients with CVD.

Our findings may also have important public health implications. The heterogeneity of treatment pathways of hypertension across data sources and countries reflects the failure of the field to converge on an effective and consistent treatment-escalation algorithm for hypertension. It is plausible that not all combination therapies have the same risks and benefits. However, current guidelines do not provide recommendations for the preferred choice of the second agent added to monotherapy, owing to the lack of evidence from RCTs,^8,17,18,23,24,25^ and the large variation observed in clinical practice may be associated with a trial-and-error approach to intensifying treatment for hypertension. This finding suggests the need to evaluate the efficacy and safety associated with different second-line antihypertensive agents. While RCTs remain a key tool for high-quality clinical efficacy estimates in controlled settings, clinical observational studies can help fill evidence gaps where large-scale RCTs are not feasible or are too costly, such as in the study of second-line and third-line antihypertensive treatments.

### Limitations

Several limitations need to be considered when interpretating the results of this study. First, our study was based on routinely collected data from clinical practice, where misclassification of diseases and therapies may be present. We included only patients who had a clinical diagnosis of hypertension; therefore, patients without a coded diagnosis would have been excluded even if they had elevated blood pressure levels that met criteria for hypertension. Second, treatment misclassification is possible given that participating data sources varied in their capture of drugs from hospital billing records, prescription orders, or dispensing data. Third, this study describes prescription patterns of antihypertensive medications, and we do not have information about medication compliance among patients with hypertension. Fourth, our study is limited to 8 countries and regions, so findings may not be generalizable to other countries.

## Conclusions

To our knowledge, this is the largest and most diverse study characterizing use of dual combination therapies in treatment escalation of hypertension in clinical practice. Large variation in drug use was observed in routine practice, suggesting the need for future research on the safety and efficacy associated with the more commonly used treatments.

## Notes

Supplement. **eAppendix 1.** Description of Data Sources **eAppendix 2.** Ethical Approval **eTable 1.** List of Included Data Sources **eTable 2.** Drug Codes of 56 Drug Ingredients in 4 Major Antihypertensive Drug Classes **eTable 3.** 12 Exposure Cohorts for Class-vs-Class Comparison **eFigure 1.** Graphical Presentation of Cohort Definitions **eFigure 2.** Forest Plots for Between-Country Heterogeneity in Treatment Use **eFigure 3.** Treatment Pathway of Hypertension in Each Database
