# Supplementary material for: Analysis of Dual Combination Therapies Used in Treatment of Hypertension in a Multinational Cohort
Source: JAMA Netw Open. 2022 Mar 24;5(3):e223877. doi: 10.1001/jamanetworkopen.2022.3877 (PMC8948532; doi:10.1001/jamanetworkopen.2022.3877)
Supplement: Supplement. — eAppendix 1. Description of Data Sources eAppendix 2. Ethical Approval eTable 1. List of Included Data Sources eTable 2. Drug Codes of 56 Drug Ingredients in 4 Major Antihypertensive Drug Classes eTable 3. 12 Exposure Cohorts for Class-vs-Class Comparison eFigure 1. Graphical Presentation of Cohort Definitions eFigure 2. Forest Plots for Between-Country Heterogeneity in Treatment Use eFigure 3. Treatment Pathway of Hypertension in Each Database [file jamanetwopen-e223877-s001.pdf]

Supplemental Online Content

Lu Y, Van Zandt M, Liu Y, et al. Analysis of dual combination therapies used in treatment of hypertension in a multinational cohort. *JAMA Netw Open*. 2022;5(3):e223877. doi:10.1001/jamanetworkopen.2022.3877

eAppendix 1. Description of Data Sources
eAppendix 2. Ethical Approval
eTable 1. List of Included Data Sources
eTable 2. Drug Codes of 56 Drug Ingredients in 4 Major Antihypertensive Drug Classes
eTable 3. 12 Exposure Cohorts for Class-vs-Class Comparison
eFigure 1. Graphical Presentation of Cohort Definitions
eFigure 2. Forest Plots for Between-Country Heterogeneity in Treatment Use
eFigure 3. Treatment Pathway of Hypertension in Each Database

This supplemental material has been provided by the authors to give readers additional information about their work.

© 2022 Lu Y et al. *JAMA Network Open.*

eAppendix 1. Description of Data Sources

IQVIA LPD Australia
The IQVIA LPD Australia database consists of anonymized patient records collected from Patient Management software used by GPs during an office visit to document patients' clinical records. The data originates from two sources: longitudinal patient data and practice profiles where they are integrated into one common data source.

IQVIA US Ambulatory EMR
The IQVIA US Ambulatory EMR database consists of longitudinal, de-identified electronic health records originating from ambulatory clients. The data contains detailed clinical information that captures important health outcomes such as lab test results and vital signs. It also covers administered drugs including prescription and over-the-counter medicines, vaccines, large-molecule biologic therapies, route of administration, days suppl and refill information.

IQVIA Longitudinal Patient Database (LPD) France
The IQVIA OMOP Longitudinal Patient Database (LPD) France database consists of anonymized patient records collected from Patient Management software used by Doctors during an office visit to document patients' clinical records. The total database consists of 1200 GPs, 7.8 million patients, 620 specialists across 8 specialties (cardiology, neurology, psychiatry, pulmonology, gastroenterology, gynecology, diabetology & rheumatology)

IQVIA LPD Italy
LPD Italy is comprised of anonymized patient records collected from software used by GPs during an office visit to document patients' clinical records. Data coverage includes over 2M patient records with at least one visit and 119.5M prescription orders across 900 GP practices. Dates of service include from 2004 through present. Observation time is defined by the first and last consultation dates. Drugs are captured as prescription records with product, quantity, dosing directions, strength, indication and date of consultation.

Khoo Teck Puat Hospital (KTPH) Singapore
KTPH is comprised of anonymized patient records collected from electronic medical records of Khoo Teck Puat Hospital, a 600-bed tertiary care facility serving the population in the northern region of Singapore. Data coverage includes approximately 1M patient records with at least one visit. Dates of service include data from 2010 onwards.

Ajou University School of Medicine (AUSOM) South Korea
The Ajou University School of Medicine (AUSOM) database consists of electronic health record data from a Korean tertiary teaching hospital with 1,108 patient beds, 33 medical departments, and 23 operating rooms. Ajou University Medical Center adopted a computerized provider order entry system in 1994 and a comprehensive electronic health record system in March 2010. The AUSOM database includes the medical records of 3.11 million patients collected from 1995-2019.

ePBRN SWSLHD 2019 Linked Dataset Australia

© 2022 Lu Y et al. *JAMA Network Open.*

The 2012-2019 Electronic Practice based Research Network 2019 Linked Dataset from South Western Sydney Local Health District (ePBRN SWSLHD) consisted of anonymised, electronic health records sourced from an integrated care network of 18 general practitioner clinics and hospitals located in South Western Sydney, New South Wales, Australia. These records are linked to hospital data using probabilistic linkage. The linked dataset covers 166,590 patients and includes patient demographics, medications, conditions, and visits to general practices and hospitals converted into OMOP-CDM.

Kyung Hee University Medical Center (KHMC) South Korea
The Kyung Hee University Medical Center (KHMC) database consists of electronic health record data from a tertiary hospital in Korea with 1,053 patient beds, 34 medical departments, and 16 operating rooms. This hospital is one of the first hospitals in Korea to attempt to computerize medical records. The KHMC database used in the current study contains medical records of 2.01 million patients collected from 2008 to 2018.

China Jiangsu Province Hospital (CJSPH)
China Jiangsu Province Hospital (CJSPH) is comprised of anonymized patient records collected from Clinical Data Repository (CDR). Data coverage includes over 6,230,000 patient records (Outpatient and Inpatient). Dates of service include from January 1st, 2005 to December 31st, 2015. Observation time is defined by the first and last visiting dates. Drugs are captured as prescription records with product, quantity, dosing directions, strength, indication and date of prescription.

National University Hospital (NUH) Singapore
National University Hospital (NUH) data consists of anonymized patient records extracted from the Electronic Health Record system of the hospital. Data coverage includes 750,270 patients across Inpatient, Outpatient and Emergency visits between 2015 to 2018.

Taipei Medical University Clinical Research Database (TMUCRD)
Taipei Medical University has been integrating the electronic medical records databases of TMU's three affiliated hospitals (Taipei Medical University Hospital, Wanfang Hospital and Shuang Ho Hospital) to form the Taipei Medical University Clinical Research Database (TMUCRD) Since 2015. It combines various electronic medical records data of the three hospitals, including structured data (such as patient's basic information, medical information, test reports, diagnosis results, treatment procedures, surgery and medication status) and unstructured data (such as physicians records, pathology reports, medical imaging reports), and compiled them into analyzable data. The data period covered by TMUCRD is from 1998 to 2020. Since Shuang Ho Hospital joined the TMU System in 2008, the scope of the database includes the complete data of the three hospitals. The data content includes 13 categories, 63 data tables and 2,506 fields. In addition, the various data tables can be mutually linked. As of 2020, the database has accumulated the medical information of nearly 3.79 million patients across Taiwan.

© 2022 Lu Y et al. *JAMA Network Open.*

eAppendix 2. Ethical Approval

All data partners received institutional review board (IRB) approval or exemption.

**Data source**	**IRB approval**
IQVIA LPD Australia	Use of de-identified IQVIA data sources was deemed not human subject research by the IQVIA internal review committee and approved for OHDSI network studies.
ePBRN SWSLHD 2019 Linked Dataset	Use of Australia ePBRN SWSLHD data source was approved by the UNSW Sydney and South Western Sydney Local Health District Human Research Ethics Committees (Project number: 2019/PID05368).
Korea Ajou University School of Medicine (AUSOM) CDM	The Korean Bioethics Act does not require an IRB review for retrospective observational studies using common data model in distributed research networks.
Korea CDM of Kyung Hee University Hospital	The Korean Bioethics Act does not require an IRB review for retrospective observational studies using common data model in distributed research networks.
Khoo Teck Puat Hospital (KTPH)	Use of KTPH data source was reviewed by National Health Group Domain Specific Review Board and approved the request for waiver of informed consent (Project number: 2017/00995).
National University Hospital (NUH)	Use of NUH data source was reviewed by National Health Group Domain Specific Review Board and was determined that it qualifies for exemption because the analysis involved a dataset without identifiers (Project number: 2021/00125).
China Jiangsu Province Hospital	Use of Jiangsu Province Hospital data source was reviewed and approved by the Nanjing Medical University Institutional Review Board.
Taiwan Taipei Medical University Clinical Research Database (TMUCRD)	Use of TMUCRD data has been approved by theTMU-Joint Institutional Review Board (Project number: TMU-JIRB N202011003).
IQVIA Ambulatory EMR	Use of de-identified IQVIA data sources was deemed not human subject research by the IQVIA internal review committee and approved for OHDSI network studies.
IQVIA LPD France	Use of de-identified IQVIA data sources was deemed not human subject research by the IQVIA internal review committee and approved for OHDSI network studies.
IQVIA LPD Italy	Use of de-identified IQVIA data sources was deemed not human subject research by the IQVIA internal review committee and approved for OHDSI network studies.

© 2022 Lu Y et al. *JAMA Network Open.*

eTable 1. List of Included Data Sources

**Data Source**	**Data Type**	**Country/District**	**Time Period**	**No. of Patients**
IQVIA LPD Australia	EHR	Australia	2006-2020	3,101,500
ePBRN SWSLHD 2019 Linked Dataset (ePBRN SWSLHD)	EHR	South Western Sydney, Australia	2012-2019	139,346
Ajou University School of Medicine (AUSOM)	EHR	Suwon, Korea	1995-2019	3,109,677
Kyung Hee University Hospital (KHMC)	EHR	Seoul, Korea	2008-2018	2,010,456
Khoo Teck Puat Hospital (KTPH)	EHR	Singapore	2010-2016	290,074
National University Hospital (NUH)	EHR	Singapore	2015-2018	750,270
China Jiangsu Province Hospital (CJSPH)	EHR	China	2005-2015	6,230,000
Taiwan Taipei Medical University Clinical Research Database (TMUCRD)	EHR	Taiwan	2004-2020	3,659,572
IQVIA US Ambulatory EMR	EHR	United States	2006-2020	78,526,000
IQVIA LPD France	EHR	France	1994-2020	18,118,000
IQVIA LPD Italy	EHR	Italy	2004-2020	2,209,600

*EHR = Electronic health record

© 2022 Lu Y et al. *JAMA Network Open.*

eTable 2. Drug Codes of 56 Drug Ingredients in 4 Major Antihypertensive Drug Classes

ACEI/ARB			Beta-blocker			Calcium channel blocker			Thiazide Diuretics		
Ingredient name	RxNorm	OMOP ID	Ingredient name	RxNorm	OMOP ID	Ingredient name	RxNorm	OMOP ID	Ingredient name	RxNorm	OMOP ID
Benazepril	18867	1335471	Esmolol	49737	19063575	Diltiazem	3443	1328165	Hydrochlorothiazide	5487	974166
Captopril	1998	1340128	Celiprolol	20498	19049145	Verapamil	11170	1307863	Xipamide	11371	19010493
Cilazapril	21102	19050216	Oxprenolol	7801	19024904	Amlodipine	17767	1332418	Chlorthalidone	2409	1395058
Enalapril	3827	1341927	Labetalol	6185	1386957	Nifedipine	7417	1318853	Indapamide	5764	978555
Fosinopril	50166	1363749	Propranolol	8787	1353766	Nicardipine	7396	1318137	Metolazone	6916	907013
Imidapril	60245	19122327	Carvedilol	20352	1346823	Felodipine	4316	1353776			
Lisinopril	29046	1308216	Pindolol	8332	1345858	Nisoldipine	7435	1319880			
Moexipril	30131	1310756	Bisoprolol	19484	1338005	Isradipine	33910	1326012			
Perindopril	54552	1373225	Penbutolol	7973	1327978	Nimodipine	7426	1319133			
Quinapril	35208	1331235	Betaxolol	1520	1322081	Clevidipine	233603	19089969			
Ramipril	35296	1334456	Acebutolol	149	1319998	Nilvadipine	53692	19113063			
Trandolapril	38454	1342439	Nebivolol	31555	1314577	Mepirodipine	39879	19102106			
Zofenopril	39990	19102107	Atenolol	1202	1314002	Manidipine	29275	19071995			
Valsartan	69749	1308842	Metoprolol	6918	1307046	Nitrendipine	7441	19020061			
Candesartan	214354	1351557	Carteolol	2116	950370	Lercanidipine	135056	19015802			
Eprosartan	83515	1346686				Lacidipine	28382	19004539			
Irbesartan	83818	1347384									
Losartan	52175	1367500									
Olmesartan	321064	40226742									
Telmisartan	73494	1317640									

OMOP ID indicates the exact code used in OMOP-CDM based on RxNorm ontology. Corresponding RxNorm code to OMOP-ID are also described in this table. ACEI = angiotensin converting enzyme inhibitor; ARB = angiotensin receptor blocker; CDM = common data model; OMOP = observational medical outcomes partnership.

eTable 3. 12 Exposure Cohorts for Class-vs-Class Comparison

Cohort #	Dual combination*
1	ACEi/ARB + Beta-blocker
2	ACEi/ARB + CCB
3	ACEi/ARB + Diuretic
4	Beta-blocker + ACEi/ARB
5	Beta-blocker + CCB
6	Beta-blocker + Diuretic
7	CCB + ACEi/ARB
8	CCB + Beta-blocker
9	CCB + Diuretic
10	Diuretic + ACEi/ARB
11	Diuretic + Beta-blocker
12	Diuretic + CCB

* ACEi/ARB + Beta-blocker denotes starting an ACEi/ARB monotherapy followed by a beta-blocker.
ACEi = angiotensin converting enzyme inhibitor; ARB = angiotensin receptor blocker; CCB = calcium channel blocker.

© 2022 Lu Y et al. *JAMA Network Open.*

eFigure 1. Graphical Presentation of Cohort Definitions

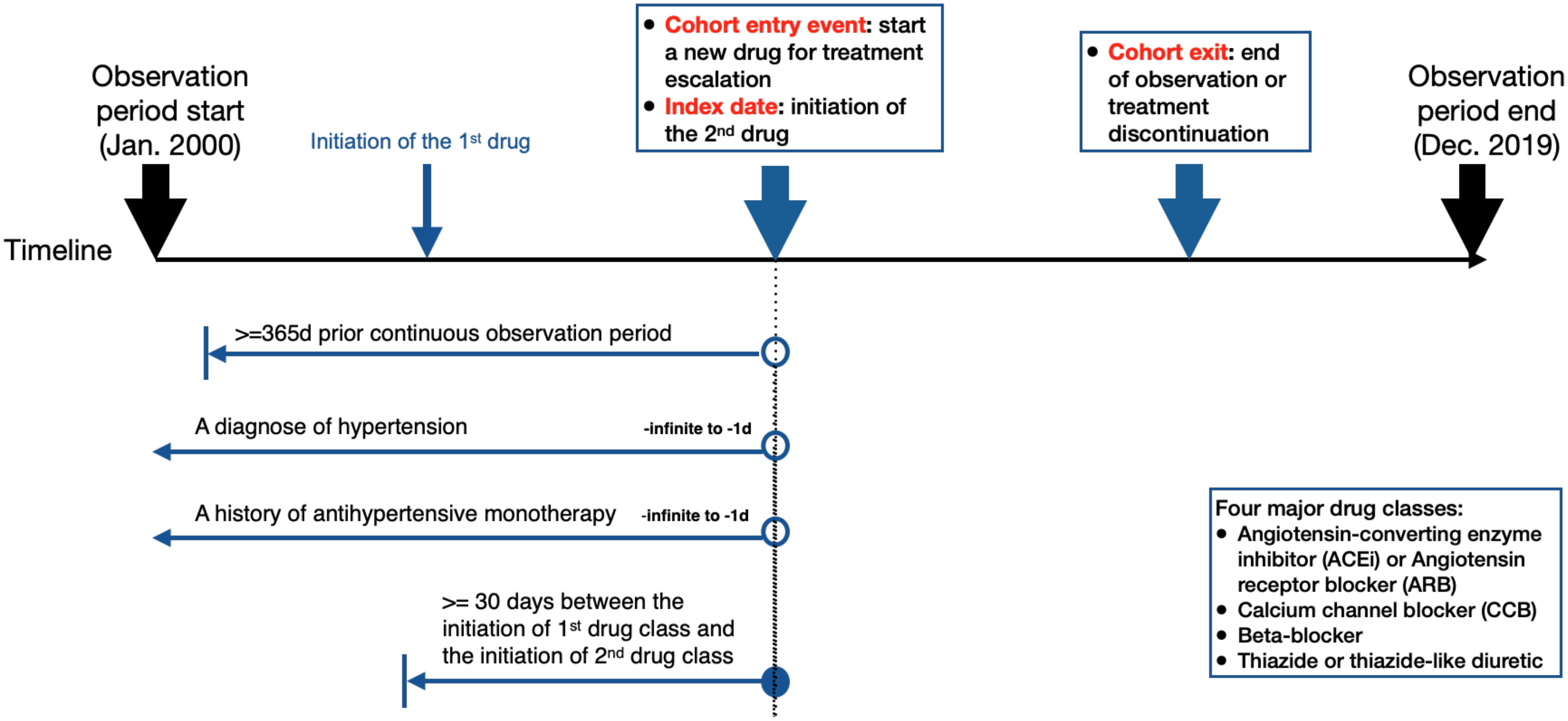

© 2022 Lu Y et al. *JAMA Network Open.*

eFigure 2. Forest Plots for Between-Country Heterogeneity in Treatment Use

(A) ACEi/ARB + CCB

Study	Events	Total	Weight	IV, Random, 95% CI
Australia	4540	11494	12.5%	0.395 [0.386; 0.404]
Korea	1363	6980	12.5%	0.195 [0.186; 0.205]
Singapore	655	2096	12.4%	0.312 [0.293; 0.333]
China	3127	7008	12.5%	0.446 [0.435; 0.458]
Taiwan	1545	8544	12.5%	0.181 [0.173; 0.189]
France	22523	103994	12.5%	0.217 [0.214; 0.219]
Italy	14628	76082	12.5%	0.192 [0.189; 0.195]
United States	95284	754137	12.5%	0.126 [0.126; 0.127]
**Total (95% CI)**		**970335**	**100.0%**	**0.251 [0.194; 0.314]**

Heterogeneity: $Tau^2 = 0.0100$; $Chi^2 = 14730.92$, df = 7 (P = 0); $I^2 = 100\%$

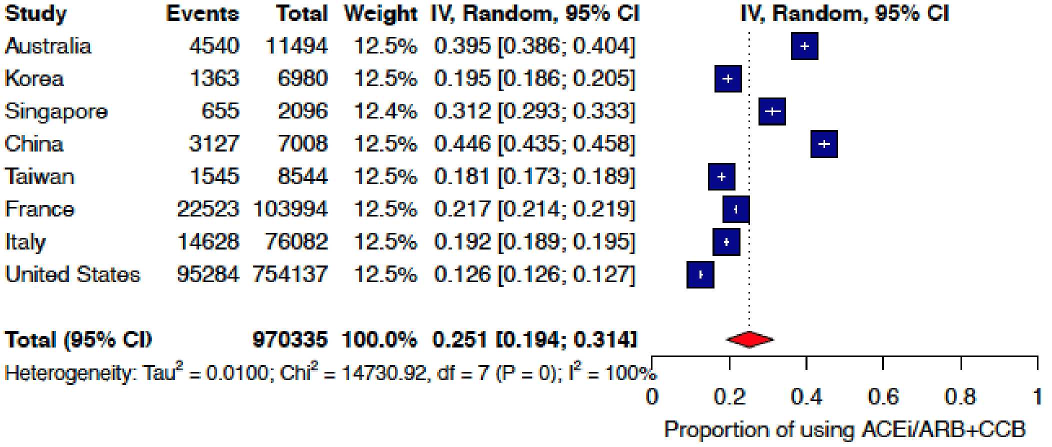

(B) CCB + ACEi/ARB

Study	Events	Total	Weight	IV, Random, 95% CI
Australia	1458	11494	12.5%	0.127 [0.121; 0.133]
Korea	1678	6980	12.5%	0.240 [0.230; 0.251]
Singapore	324	2096	12.4%	0.155 [0.139; 0.171]
China	3312	7008	12.5%	0.473 [0.461; 0.484]
Taiwan	2651	8544	12.5%	0.310 [0.300; 0.320]
France	15749	103994	12.5%	0.151 [0.149; 0.154]
Italy	5841	76082	12.5%	0.077 [0.075; 0.079]
United States	54297	754137	12.5%	0.072 [0.071; 0.073]
**Total (95% CI)**		**970335**	**100.0%**	**0.187 [0.133; 0.249]**

Heterogeneity: $Tau^2 = 0.0115$; $Chi^2 = 16907.45$, df = 7 (P = 0); $I^2 = 100\%$

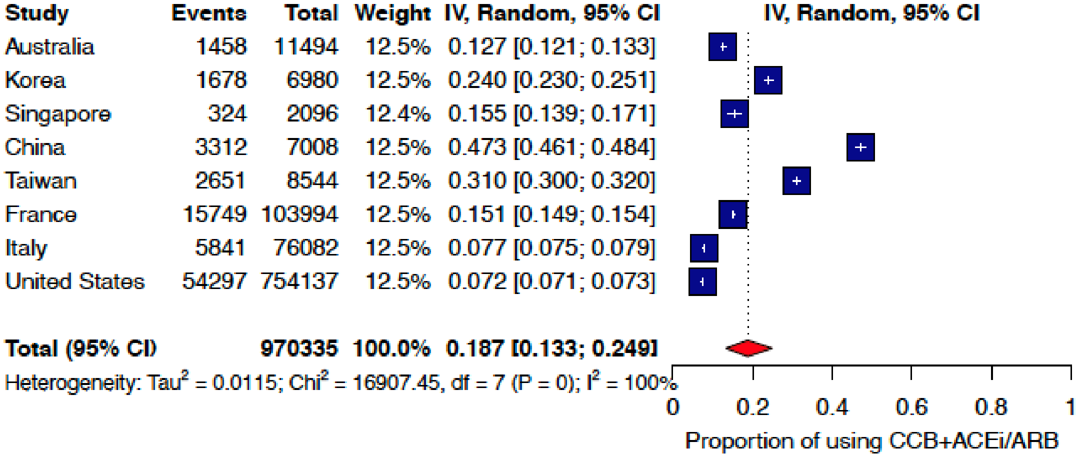

© 2022 Lu Y et al. *JAMA Network Open.*

(C) ACEi/ARB + Diuretic

Study	Events	Total	Weight	IV, Random, 95% CI
Australia	2350	11494	12.5%	0.204 [0.197; 0.212]
Korea	486	6980	12.5%	0.070 [0.064; 0.076]
Singapore	47	2096	12.4%	0.022 [0.017; 0.030]
China	111	7008	12.5%	0.016 [0.013; 0.019]
Taiwan	3	8544	12.5%	0.000 [0.000; 0.001]
France	22399	103994	12.5%	0.215 [0.213; 0.218]
Italy	16988	76082	12.5%	0.223 [0.220; 0.226]
United States	123940	754137	12.5%	0.164 [0.164; 0.165]
**Total (95% CI)**		**970335**	**100.0%**	**0.089 [0.056; 0.129]**

Heterogeneity: $Tau^2 = 0.0085$; $Chi^2 = 12434.35$, df = 7 (P = 0); $I^2 = 100\%$

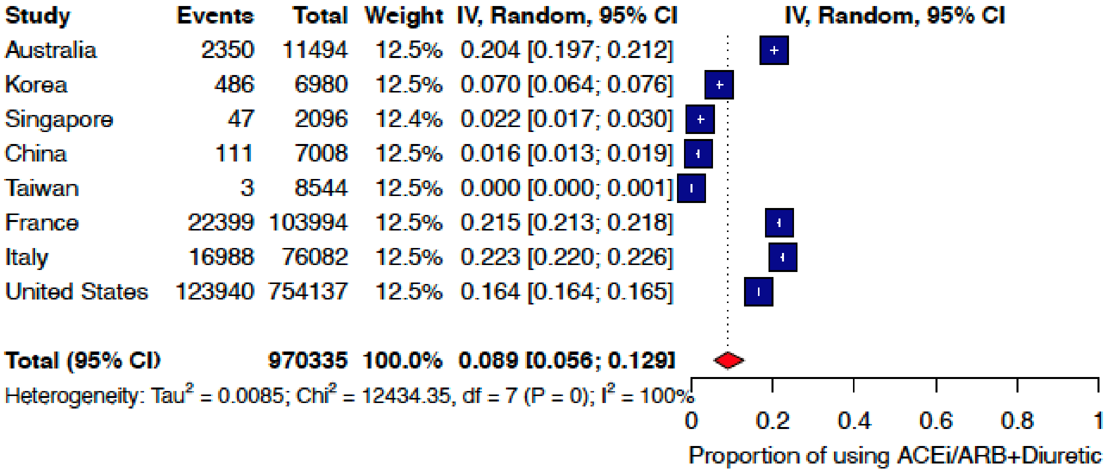

(D) Diuretic + ACEi/ARB

Study	Events	Total	Weight	IV, Random, 95% CI
Australia	300	11494	12.5%	0.026 [0.023; 0.029]
Korea	156	6980	12.5%	0.022 [0.019; 0.026]
Singapore	15	2096	12.4%	0.007 [0.004; 0.012]
China	114	7008	12.5%	0.016 [0.013; 0.020]
Taiwan	0	8544	12.5%	0.000 [0.000; 0.000]
France	3281	103994	12.5%	0.032 [0.030; 0.033]
Italy	5749	76082	12.5%	0.076 [0.074; 0.077]
United States	84275	754137	12.5%	0.112 [0.111; 0.112]
**Total (95% CI)**		**970335**	**100.0%**	**0.027 [0.009; 0.057]**

Heterogeneity: $Tau^2 = 0.0113$; $Chi^2 = 16590.15$, df = 7 (P = 0); $I^2 = 100\%$

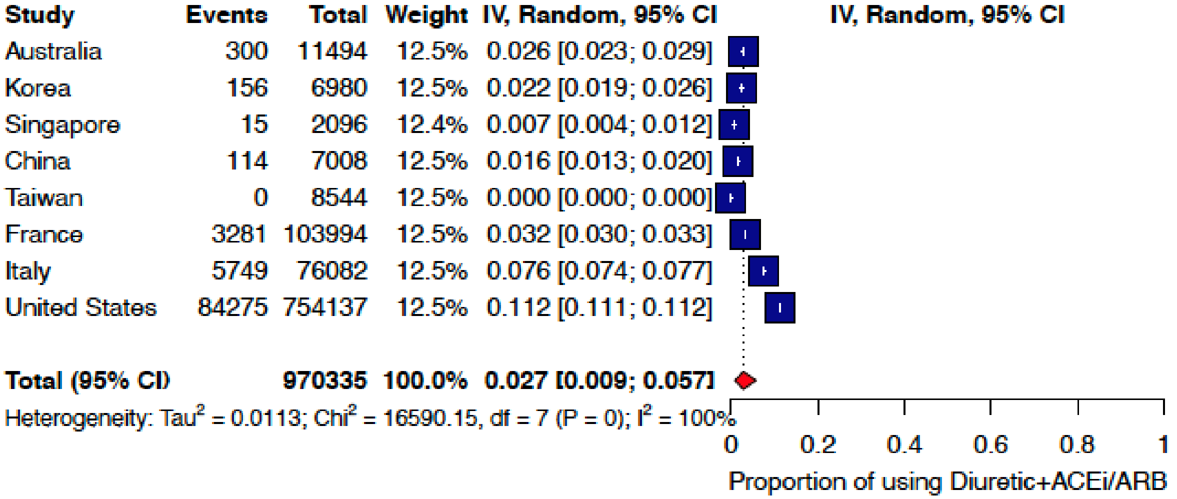

© 2022 Lu Y et al. *JAMA Network Open.*

(E) ACEi/ARB + Beta-blocker

Study	Events	Total	Weight	IV, Random, 95% CI
Australia	1346	11494	12.5%	0.117 [0.111; 0.123]
Korea	441	6980	12.5%	0.063 [0.058; 0.069]
Singapore	249	2096	12.2%	0.119 [0.105; 0.133]
China	46	7008	12.5%	0.007 [0.005; 0.009]
Taiwan	748	8544	12.5%	0.088 [0.082; 0.094]
France	11236	103994	12.6%	0.108 [0.106; 0.110]
Italy	11844	76082	12.6%	0.156 [0.153; 0.158]
United States	110579	754137	12.6%	0.147 [0.146; 0.147]
**Total (95% CI)**		**970335**	**100.0%**	**0.092 [0.071; 0.117]**

Heterogeneity: $Tau^2 = 0.0032$; $Chi^2 = 4719.26$, df = 7 (P = 0); $I^2 = 100\%$

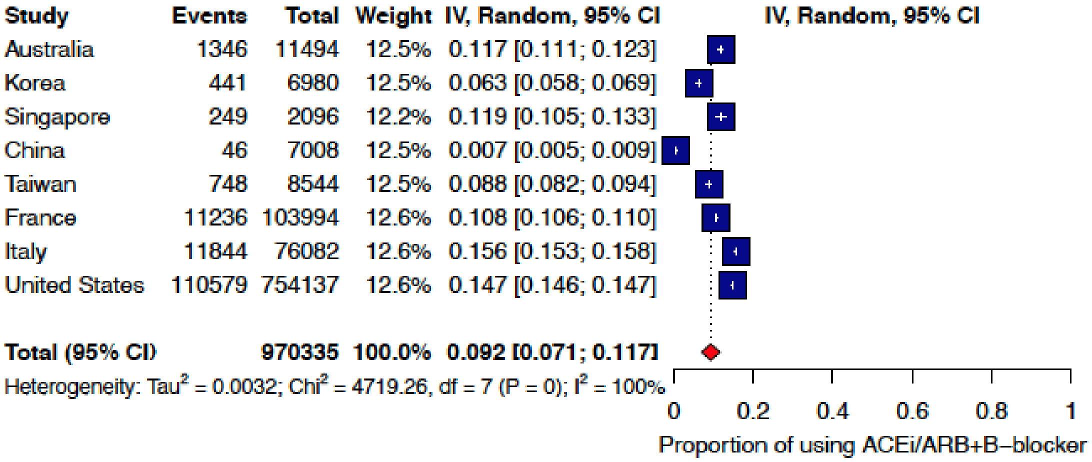

(F) Beta-blocker + ACEi/ARB

Study	Events	Total	Weight	IV, Random, 95% CI
Australia	845	11494	12.5%	0.074 [0.069; 0.078]
Korea	484	6980	12.5%	0.069 [0.063; 0.076]
Singapore	196	2096	12.2%	0.094 [0.081; 0.107]
China	26	7008	12.5%	0.004 [0.002; 0.005]
Taiwan	1250	8544	12.5%	0.146 [0.139; 0.154]
France	11116	103994	12.6%	0.107 [0.105; 0.109]
Italy	8264	76082	12.6%	0.109 [0.106; 0.111]
United States	106380	754137	12.6%	0.141 [0.140; 0.142]
**Total (95% CI)**		**970335**	**100.0%**	**0.085 [0.063; 0.109]**

Heterogeneity: $Tau^2 = 0.0034$; $Chi^2 = 5029.71$, df = 7 (P = 0); $I^2 = 100\%$

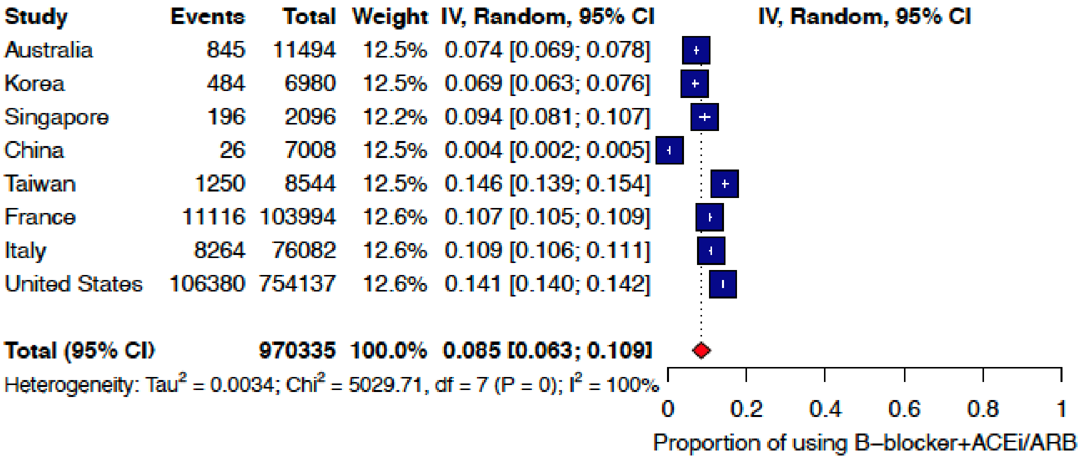

© 2022 Lu Y et al. *JAMA Network Open.*

(G) CCB + Diuretic

Study	Events	Total	Weight	IV, Random, 95% CI
Australia	92	11494	12.6%	0.008 [0.006; 0.010]
Korea	274	6980	12.5%	0.039 [0.035; 0.044]
Singapore	17	2096	11.8%	0.008 [0.005; 0.013]
China	78	7008	12.5%	0.011 [0.009; 0.014]
Taiwan	1	8544	12.5%	0.000 [0.000; 0.001]
France	1660	103994	12.7%	0.016 [0.015; 0.017]
Italy	1103	76082	12.7%	0.014 [0.014; 0.015]
United States	21108	754137	12.7%	0.028 [0.028; 0.028]
**Total (95% CI)**		**970335**	**100.0%**	**0.013 [0.008; 0.020]**

Heterogeneity: $Tau^2 = 0.0015$; $Chi^2 = 2228.49$, df = 7 (P = 0); $I^2 = 100\%$

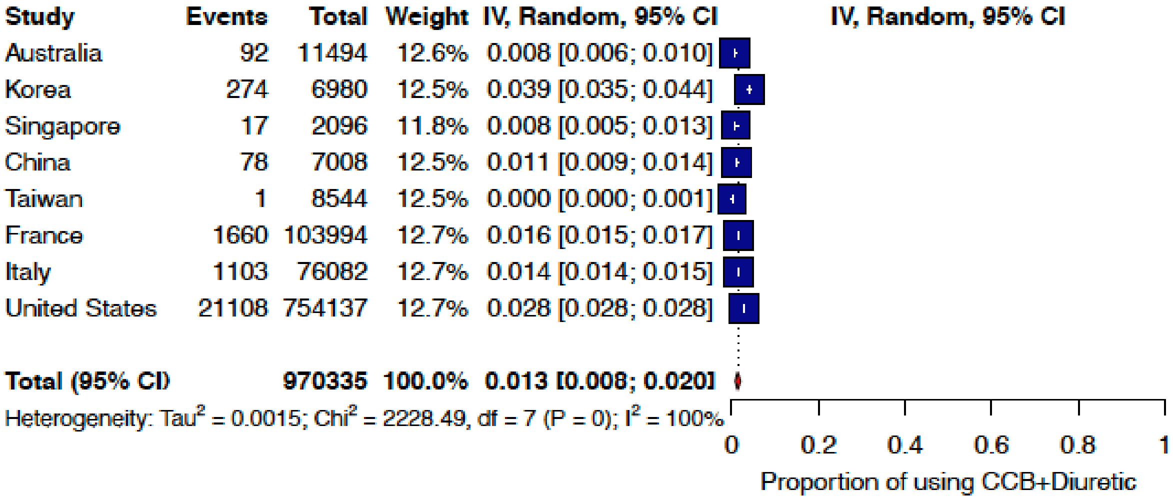

(H) Diuretic + CCB

Study	Events	Total	Weight	IV, Random, 95% CI
Australia	67	11494	12.5%	0.006 [0.005; 0.007]
Korea	145	6980	12.5%	0.021 [0.018; 0.024]
Singapore	11	2096	12.0%	0.005 [0.003; 0.009]
China	140	7008	12.5%	0.020 [0.017; 0.024]
Taiwan	0	8544	12.5%	0.000 [0.000; 0.000]
France	1097	103994	12.7%	0.011 [0.010; 0.011]
Italy	1539	76082	12.7%	0.020 [0.019; 0.021]
United States	22568	754137	12.7%	0.030 [0.030; 0.030]
**Total (95% CI)**		**970335**	**100.0%**	**0.012 [0.006; 0.020]**

Heterogeneity: $Tau^2 = 0.0022$; $Chi^2 = 3238.48$, df = 7 (P = 0); $I^2 = 100\%$

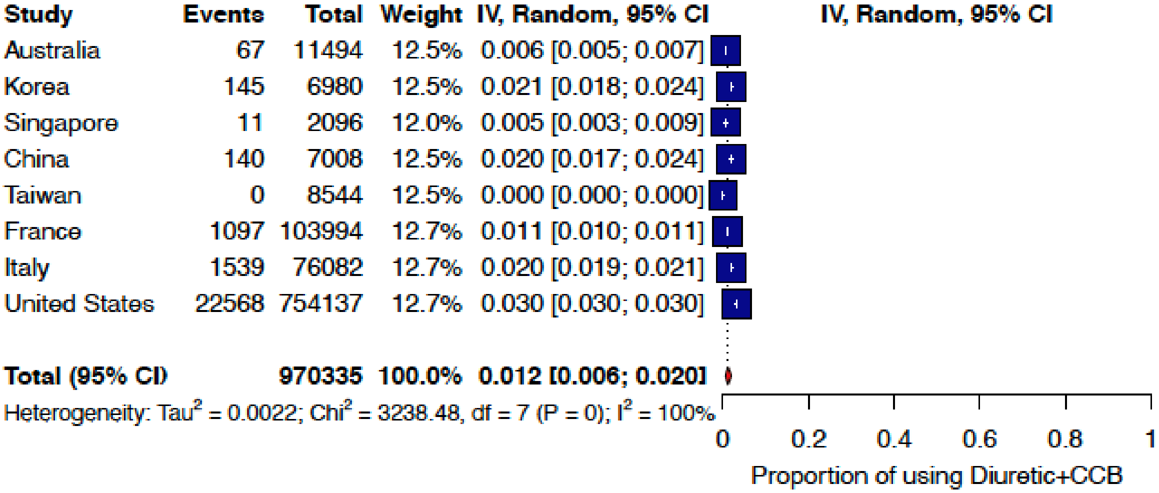

© 2022 Lu Y et al. *JAMA Network Open.*

(J) CCB + Beta-blocker

Study	Events	Total	Weight	IV, Random, 95% CI
Australia	219	11494	12.6%	0.019 [0.017; 0.022]
Korea	1031	6980	12.5%	0.148 [0.139; 0.156]
Singapore	221	2096	12.0%	0.105 [0.093; 0.119]
China	34	7008	12.5%	0.005 [0.003; 0.007]
Taiwan	1182	8544	12.5%	0.138 [0.131; 0.146]
France	3866	103994	12.7%	0.037 [0.036; 0.038]
Italy	2475	76082	12.7%	0.033 [0.031; 0.034]
United States	30593	754137	12.7%	0.041 [0.040; 0.041]
**Total (95% CI)**		**970335**	**100.0%**	**0.055 [0.041; 0.070]**

Heterogeneity: $Tau^2 = 0.0021$; $Chi^2 = 3100.88$, df = 7 (P = 0); $I^2 = 100\%$

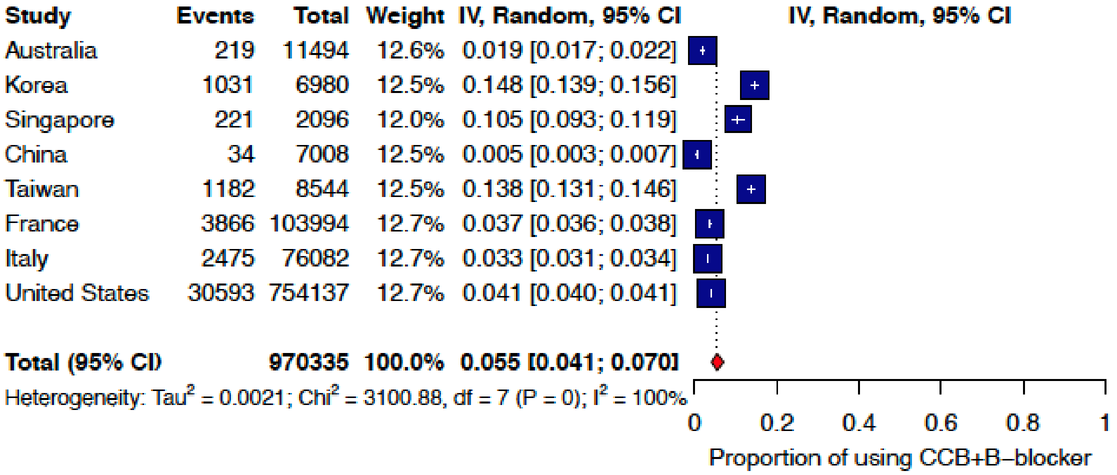

(K) Beta-blocker + CCB

Study	Events	Total	Weight	IV, Random, 95% CI
Australia	199	11494	12.5%	0.017 [0.015; 0.020]
Korea	813	6980	12.5%	0.116 [0.109; 0.124]
Singapore	340	2096	12.0%	0.162 [0.147; 0.179]
China	19	7008	12.5%	0.003 [0.002; 0.004]
Taiwan	1163	8544	12.5%	0.136 [0.129; 0.144]
France	5972	103994	12.7%	0.057 [0.056; 0.059]
Italy	2755	76082	12.6%	0.036 [0.035; 0.038]
United States	41388	754137	12.7%	0.055 [0.054; 0.055]
**Total (95% CI)**		**970335**	**100.0%**	**0.060 [0.046; 0.077]**

Heterogeneity: $Tau^2 = 0.0023$; $Chi^2 = 3339.19$, df = 7 (P = 0); $I^2 = 100\%$

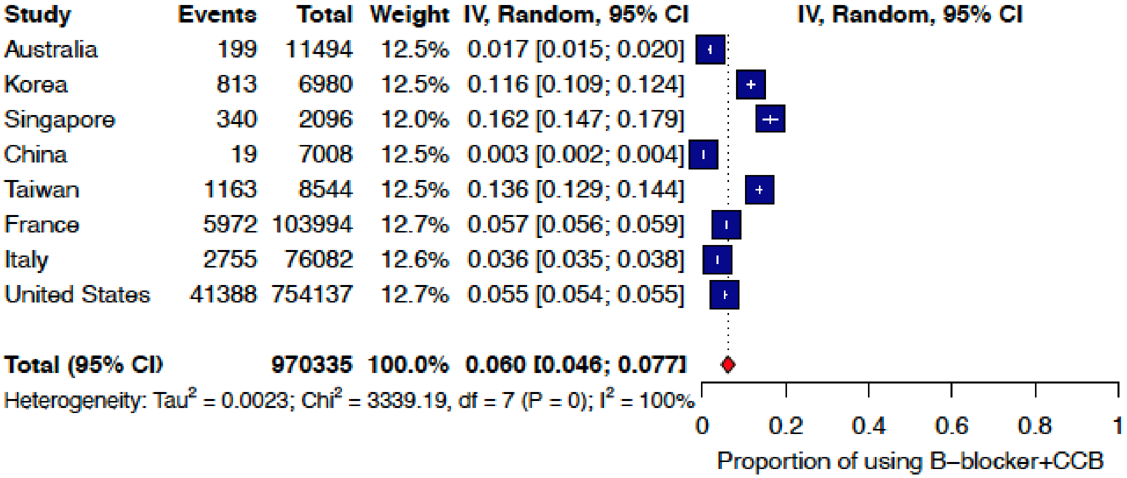

© 2022 Lu Y et al. *JAMA Network Open.*

(L) Diuretic + Beta-blocker

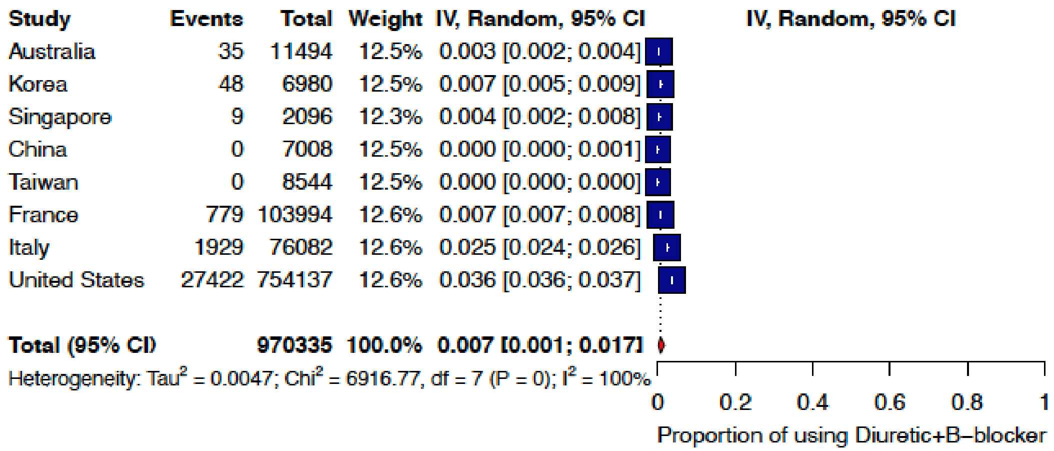

(M) Beta-blocker + Diuretic

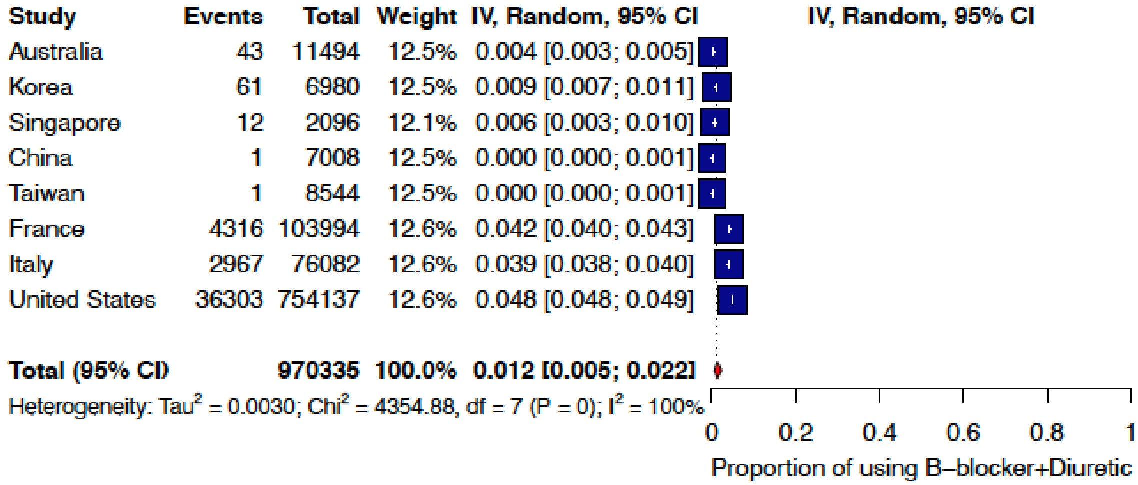

© 2022 Lu Y et al. *JAMA Network Open.*

eFigure 3. Treatment Pathway of Hypertension in Each Database

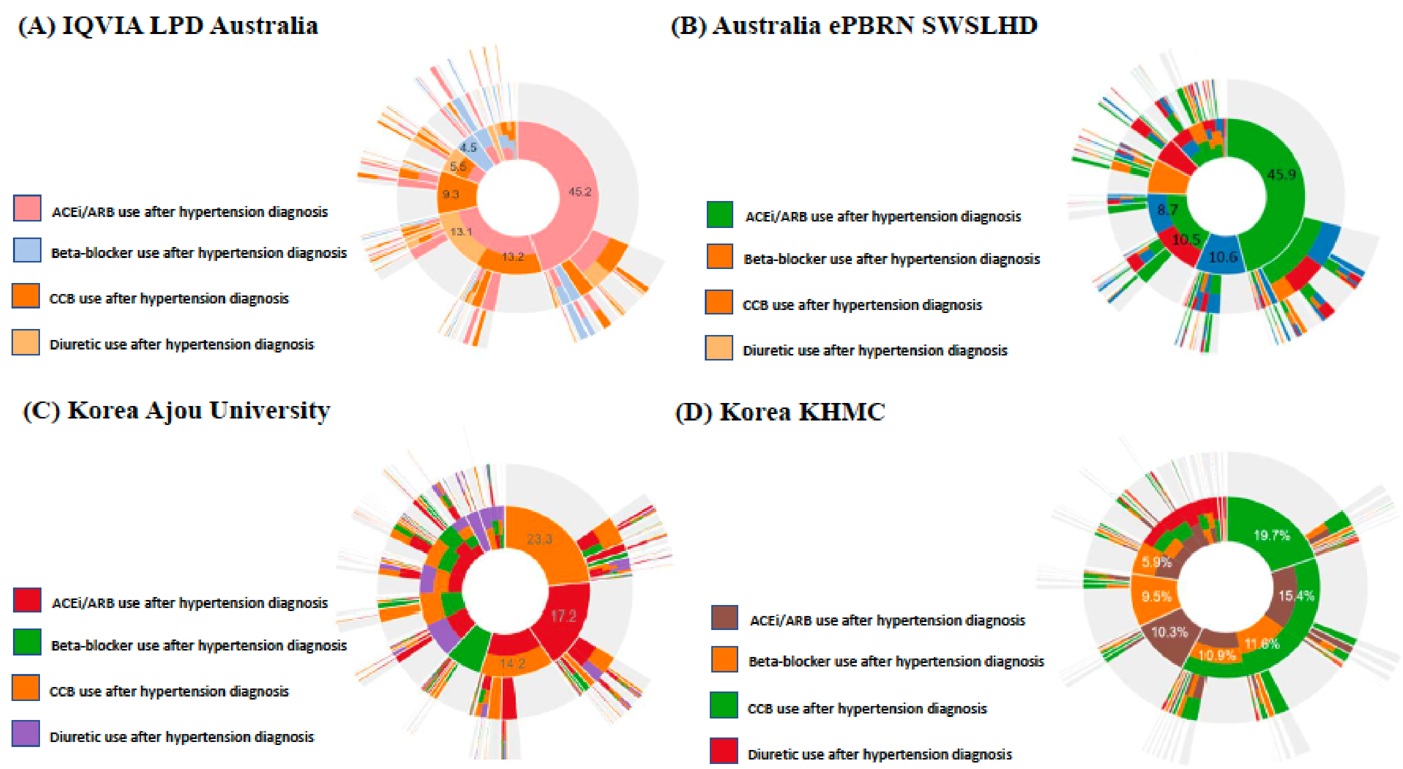

© 2022 Lu Y et al. *JAMA Network Open.*

(E) Singapore KTPH

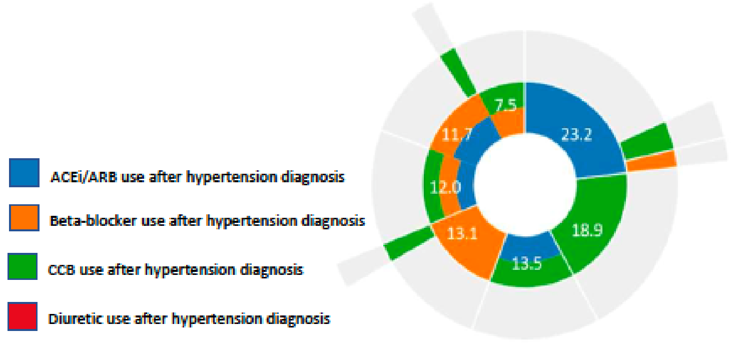

(F) Singapore NUH

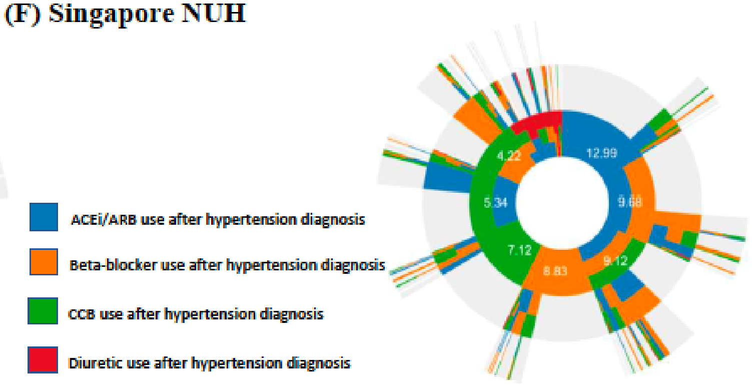

(G) China Jiangsu Province Hospital

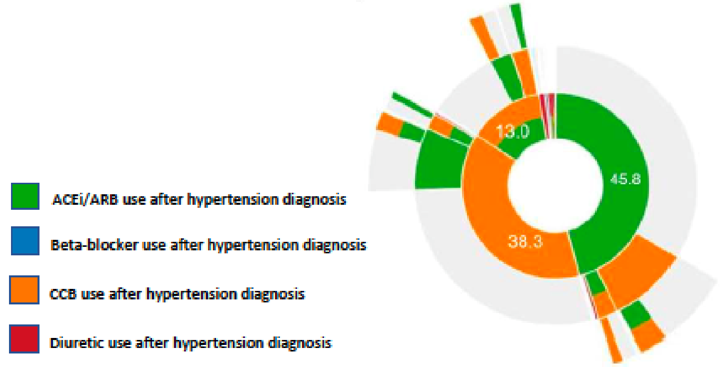

(H) Taiwan TMUCRD

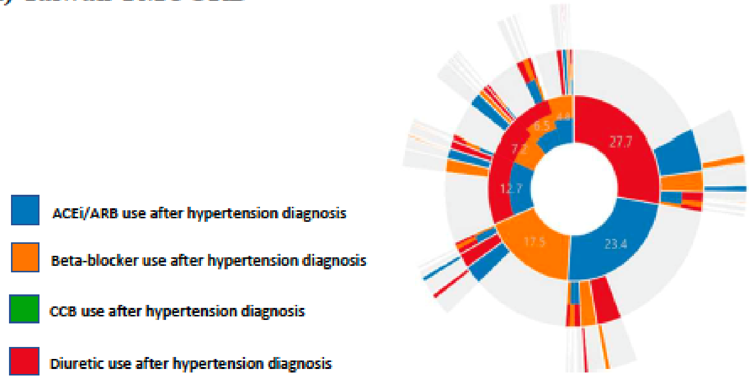

© 2022 Lu Y et al. *JAMA Network Open.*

(I) IQVIA LPD France

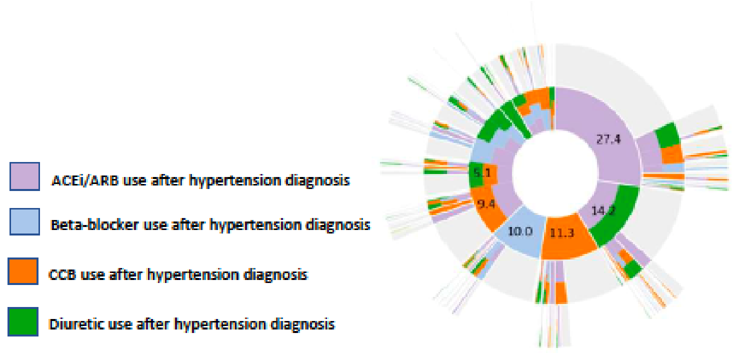

(J) IQVIA Italy LPD

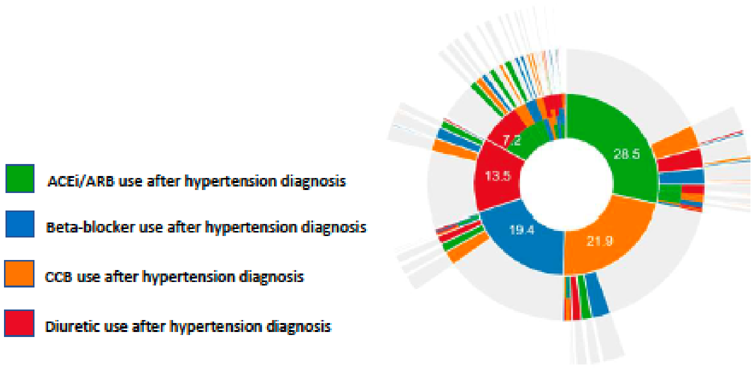

(K) IQVIA US AmbEMR

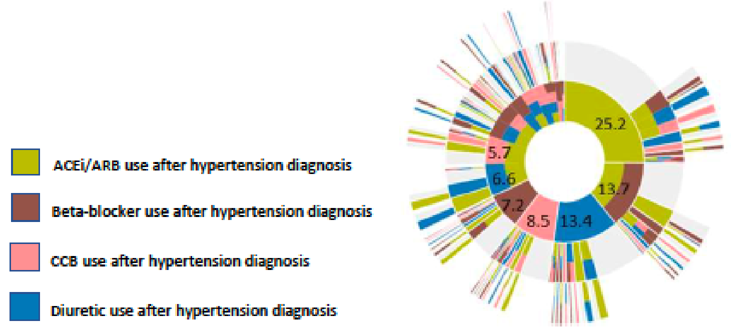

© 2022 Lu Y et al. *JAMA Network Open.*
